# Droplet Generation and Manipulation in Microfluidics: A Comprehensive Overview of Passive and Active Strategies

**DOI:** 10.3390/bios15060345

**Published:** 2025-05-29

**Authors:** Andrea Fergola, Alberto Ballesio, Francesca Frascella, Lucia Napione, Matteo Cocuzza, Simone Luigi Marasso

**Affiliations:** 1 Department of Applied Science and Technology (DISAT), Politecnico di Torino, 10129 Torino, Italy; andrea.fergola@polito.it (A.F.); francesca.frascella@polito.it (F.F.); lucia.napione@polito.it (L.N.); matteo.cocuzza@polito.it (M.C.); simone.marasso@polito.it (S.L.M.); 2 Consiglio Nazionale delle Ricerche, Istituto dei Materiali per l’Elettronica ed il Magnetismo (CNR IMEM), 43124 Parma, Italy

**Keywords:** droplet, microfluidics, trapping

## Abstract

Droplet-based microfluidics (DBM) has emerged as a powerful tool for a wide range of biochemical applications, from single-cell analysis and drug screening to diagnostics and tissue engineering. This review provides a comprehensive overview of the latest advancements in droplet generation and trapping techniques, highlighting both passive and active approaches. Passive methods—such as co-flow, cross-flow, and flow-focusing geometries—rely on hydrodynamic instabilities and capillary effects, offering simplicity and integration with compact devices, though often at the cost of tunability. In contrast, active methods exploit external fields—electric, magnetic, thermal, or mechanical—to enable on-demand droplet control, allowing for higher precision and throughput. Furthermore, we explore innovative trapping mechanisms such as hydrodynamic resistance networks, microfabricated U-shaped wells, and anchor-based systems that enable precise spatial immobilization of droplets. In the final section, we also examine active droplet sorting strategies, including electric, magnetic, acoustic, and thermal methods, as essential tools for downstream analysis and high-throughput workflows. These manipulation strategies facilitate in situ chemical and biological analyses, enhance experimental reproducibility, and are increasingly adaptable to industrial-scale applications. Emphasis is placed on the design flexibility, scalability, and biological compatibility of each method, offering critical insights for selecting appropriate techniques based on experimental needs and operational constraints.

## 1. Introduction

Over the past decades, microfluidics has become an essential tool across diverse fields, including drug discovery [1,2,3,4,5], single-cell research [6,7,8,9,10,11,12], medical diagnostics (such as point-of-care technologies) [13,14,15,16,17,18,19,20,21], and tissue engineering [22,23,24]. A key subset of microfluidics is droplet-based microfluidics (DBM), which focuses on creating discrete fluid volumes using immiscible phases, such as oil-in-water (O/W) or water-in-oil (W/O) droplets [25,26]. This approach offers significant advantages, including precise control over small fluid volumes, high-throughput experimentation, enhanced mixing efficiency, a high surface-area-to-volume ratio, and accelerated reaction rates [27,28].

DBM has found extensive applications in chemical and biomedical engineering [29,30], providing versatile platforms and techniques [31]. For instance, droplets in DBM serve as templates for single-cell analyses [32,33], polymerase chain reaction (PCR) assays [34,35,36,37], organ-on-a-chip studies [38,39,40], reagent mixing [41], and the detection of biologically active compounds [42]. In these applications, droplets act as microreactors, which can be routed or immobilized in designated traps for detailed analysis.

A major strength of DBM lies in its ability to generate highly uniform (monodisperse) droplets and encapsulate substances with precision [31,43]. However, a key challenge remains: the selective manipulation and immobilization of individual droplets from a large population under specific conditions. Recent technological advancements have addressed this issue, enabling high-efficiency droplet transportation [44,45], merging [46], dispersion [47], trapping [48], and sorting at remarkably high throughput [49,50,51], thereby expanding the potential of DBM in both research and industrial applications. Among these techniques, droplet sorting is particularly critical during screening procedures. Its purpose is to isolate desired droplets for further use.

Droplet sorting employs various methods, including pneumatic [52], magnetic [53], thermal [54], acoustic [55], and electric approaches [51]. The most widely used technique is dielectrophoresis (DEP), where droplets are deflected by an electric field into specific channels. Other methods involve pneumatic valves and external acoustic waves. When combined with rapid analytical assessments, these sorting techniques enable high throughput hit identification and downstream analysis, enhancing both research and industrial applications [56].

Over the years, several excellent reviews have explored droplet microfluidics. These works range from concise overviews of droplet generation techniques [57] and single-cell encapsulation methods [58] to comprehensive studies on passive microfluidic techniques [59] and electrowetting-based [60] manipulations and challenges in integrating biology, chemistry, and physics in assays [61,62]. They also address topics like the selection of optimal surfactants [63] and provide extensive surveys of the field through detailed review articles and books [64,65].

This review focuses on recently developed techniques for manipulating droplets in microchannels, encompassing both active and passive methods. It aims to offer an impartial evaluation of each technique’s advantages and limitations, based on available disclosures from researchers. While some of these methods remain in the academic research phase, awaiting practical and commercial adoption, their potential for transformative applications is undeniable.

This paper is organized as follows: after introducing the field of droplet manipulation in microfluidics and underscoring the importance of selective extraction in biological and engineering applications, we present a detailed examination of droplet generation methods, including passive and active approaches and their relevance to encapsulation and single-cell analysis. Advanced techniques for droplet trapping, selection, and sorting are then reviewed, focusing on both traditional and emerging methods.

### 1.1. Droplet Generation Methods

In the realm of droplet generation, microfluidic devices offer numerous advantages, enabling precise control over generation parameters, such as droplet size and internal composition. Furthermore, due to their flexibility and versatility, these devices can be adapted to meet specific experimental needs, opening new perspectives for encapsulating substances or cells in microscale droplets. In this section, we will explore the key techniques and approaches used in the design and realization of microfluidic devices for droplet generation.

In the context of microfluidic devices, droplet generation occurs by introducing a dispersed flow within a continuous flow [66]. This process exploits the fluid instability, particularly at the interface between two immiscible phases. Although various methods have been developed to introduce the dispersed phase into the continuous one and generate droplets, the fluid behavior can be characterized through key dimensionless numbers, which are calculated based on fluid properties, flow conditions, and geometrical parameters.

**Figure 1:**
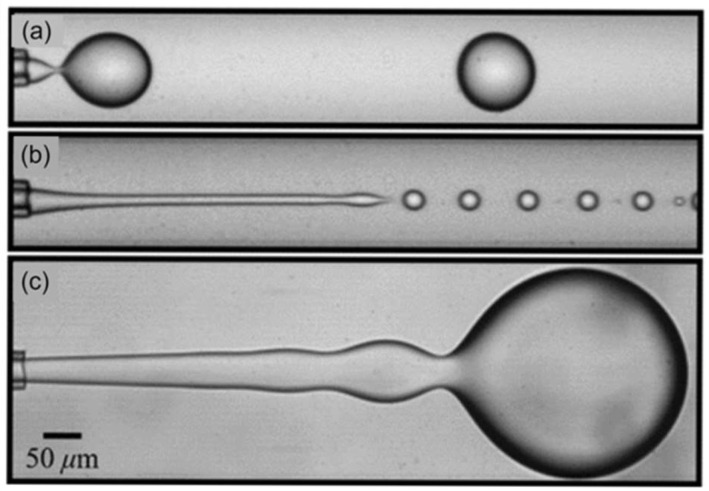
Different droplet generation regimes in the co-flow method: (**a**) dripping regime, (**b**) narrow jetting regime, and (**c**) wide jetting regime. Figure (**a**–**c**) reprinted with permission from Ref. [70]. Copyright 2007 by American Chemical Society.

The main parameter governing droplet formation is the capillary number (Ca), which quantifies the ratio between viscous forces and interfacial tension during flow. As Ca increases, the flow regimes in droplet generation transition from dripping to jetting, the latter including both narrowing and widening jet modes. For a more detailed discussion of these regimes, readers are referred to comprehensive reviews [67,68]. Figure 1 illustrates this transition within a co-flow device, highlighting the progression from the dripping regime to an extended jetting regime [69].

In addition, there are also some other dimensionless numbers which are related to the droplet breakup under a high flow rate or a large-dimensional geometry. For example, Weber number (We) is used to report the relative importance of inertia with respect to interfacial tension; Bond number (Bo) reflects the relative importance of gravitational forces with reference to interfacial tension; and Reynolds number (Re) indicates the relative importance of inertial forces with respect to viscous forces. The computational formulas and physical significance of these dimensionless numbers are summarized in Table 1.

#### 1.1.1. Droplet Generation by Passive Methods

Microfluidic droplet generators are classified into two main categories, namely passive and active systems, according to the actuation mechanisms that underpin their operation. In passive systems, fluid flow is driven by external pumps, such as syringe or pressure pumps. These systems exploit intrinsic forces, such as hydrodynamic instabilities or capillary action, to facilitate droplet formation. Passive methods generally offer lower precision in controlling droplet size and generation frequency compared to active techniques. However, they remain attractive for their relative simplicity and compatibility with compact chip designs.

In passive systems, which are typically powered by external pumps, two immiscible fluids—the dispersed phase and the continuous phase—converge at a junction, where the immiscible interface undergoes deformation and eventual droplet breakup.

**Figure 2:**
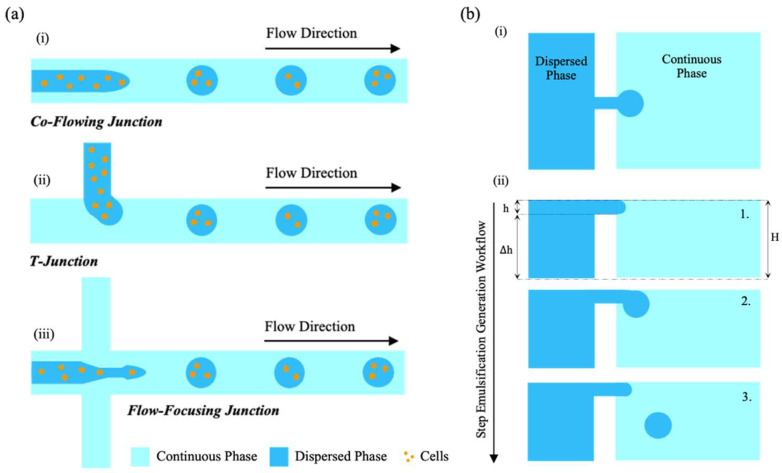
(**a**) Schematics of the generation of cell-laden hydrogel microcapsules by droplet-based microfluidics. There are three basic types of microfluidic devices for cell encapsulation: (**i**) coaxial; (**ii**) T-junction; and (**iii**) flow-focusing junction. (**b**) Schematic representation of the step emulsification mechanism: (**i**) Top view of the microfluidic architecture, showing the dispersed phase flowing through a shallow nozzle into a deep reservoir filled with the continuous phase. (**ii**) Side view of the microfluidic architecture, showing cross-sectional sequence of droplet formation in two lateral directions.

According to the complex geometrical design of the microchannel junction, the four most common microfluidic geometries used in passive droplet generation are co-flow, cross-flow, flow-focusing, and step emulsification. Schematic illustrations of droplet generations with different microchannel geometries are shown in Figure 2a, while a representative example of step emulsification is illustrated in Figure 2b. In contrast to the shear-based mechanisms of the first three, step emulsification exploits a sudden change in channel depth to induce capillary pressure-driven droplet breakup, yielding highly monodisperse droplets largely independent of flow rate [71].

Beyond these standard geometries, alternative strategies have also been explored; for example, Häberle et al. introduced an innovative approach utilizing a rotating microfluidic device to generate centripetal force for droplet production within a traditional flow-focusing geometry [72]. This method is particularly noteworthy because it eliminates the need for external pumps, demonstrating that such pumps are not the only means of creating the pressure gradient required for droplet generation. This opens up alternative strategies for pressure-driven microfluidic systems, offering potential for simpler and more portable designs. Furthermore, other pumpless methodologies could similarly be employed for the same purpose, as highlighted in recent advancements in capillary-driven microfluidics. For instance, deterministic lateral displacement (DLD) has been successfully implemented in capillary-driven systems, achieving high separation efficiencies for particles and cells without external power sources [73,74]. These approaches underscore the versatility and potential of pumpless systems in microfluidics, paving the way for cost-effective and user-friendly point-of-care devices.

#### 1.1.2. Co-Flow Geometry

In the co-flow configuration, two immiscible phases flow in the same direction through a set of coaxial microchannels. The dispersed phase is introduced via an inner channel, while the continuous phase flows through an outer concentric channel, as illustrated in Figure 2a(i) [75]. This concept, initially proposed by Umbanhowar et al. [76], can be implemented using either a quasi-two-dimensional planar device or a three-dimensional coaxial device. In these setups, the dispersed and continuous phases are aligned into parallel flows, creating a stable immiscible interface for droplet generation.

The main parameter influencing droplet size in co-flow systems is the flow rate ratio (QdQc) which determines whether the system operates in a dripping or jetting regime.

In the dripping regime, droplet detachment occurs near the nozzle due to interfacial tension overcoming shear stress. In the jetting regime, a stable thread forms and breaks downstream due to Rayleigh–Plateau instabilities (see Figure 1). In contrast to other systems such as flow-focusing systems, co-flow geometries do not rely on geometric constriction, but rather on viscous forces along the flow direction, making them particularly suitable for high-viscosity systems or when working with fragile biological samples.

Channel dimensions, especially the inner capillary diameter, directly influence the minimal achievable droplet size. Moreover, co-flow devices exhibit a reduced dependence on channel surface wettability, making them robust in systems where surface treatment is challenging.

Co-flow geometry has been widely employed for biocompatible emulsification, such as single-cell encapsulation, due to its low shear stress and gentle flow conditions. It is also particularly suitable for producing core–shell droplets, double emulsions, and monodisperse particles in pharmaceutical formulation, nutraceutical delivery, and materials chemistry.

#### 1.1.3. Cross-Flow Geometry (T-Junction)

The cross-flow configuration is achieved using angled microchannels, where the continuous and dispersed phases meet at an angle ranging from 0° to 180°. Among these, the T-junction, where the phases intersect perpendicularly, is the most common and historically the first cross-flow geometry developed for droplet generation, as shown in Figure 2a(ii) [75]. This setup induces perpendicular shear stress at the interface of the two immiscible phases, introducing nonlinearity and instability that facilitate droplet formation.

Among cross-flow devices, T-junction geometries are particularly favored for their simplicity and their ability to produce highly monodisperse droplets, achieving a coefficient of variation (CV) as low as 2%. Such characteristics make cross-flow geometries highly suitable for applications like single-cell analysis, particularly as further advancements enhance droplet uniformity [77,78].

Xu et al. demonstrated that the hydrodynamic regime in a T-junction is influenced by interfacial instability, the wetting properties of the materials, the viscous forces in the continuous phase and the interfacial tension between the two immiscible flows [79]. They developed an empirical equation to estimate droplet volume:

(1) $$V_{d}\left(\mu l\right)=0.024{\left(\frac{Q_{d}}{Q_{c}}\right)}^{-0.5}$$

where V_d_ is the volume of the droplet, Q_c_ is the volumetric flow rate of the dispersed phase, and Q_c_ is the volumetric flow rate of the continuous phase. Meanwhile, the droplet diameter D_d_ can also be estimated as D_i_/Ca, where D_i_ is the hydraulic diameter at the T-junction, and Ca is the capillary number. This highlights that droplet size correlates positively with Q_d_ and the viscosity of the continuous phase.

#### 1.1.4. Flow-Focusing Geometry

The flow-focusing geometry consists of three channels: a main channel and two symmetric side channels. These channels converge at a narrow constriction that connects to the downstream channel. In this region, the two immiscible phases flow coaxially, and the narrowing serves as a shear-focusing mechanism, promoting the formation of uniform droplets, as illustrated in Figure 2a(iii) [75].

This configuration is particularly effective for producing relatively small droplets, with the droplet generation process closely tied to the dimensions of the narrow region. An advantage of this design is that the dispersed phase experiences only the driving force within the focused zone. The symmetry of the side channels ensures that forces from opposing directions cancel out, minimizing disturbances to encapsulated cells and enhancing droplet stability.

Droplet size in flow-focusing geometries results from a complex balance of interfacial, viscous, and wetting forces, rather than being determined solely by the flow rate ratio between the dispersed and continuous phases. While an increased continuous-phase flow rate relative to the dispersed phase generally promotes the formation of smaller droplets, this effect is modulated by the viscosity of the fluids, the wetting behavior of the channel surfaces, and the geometry of the focusing region. For instance, when the dispersed phase preferentially wets the channel walls, long slug-like structures tend to form. In contrast, when the continuous phase exhibits strong non-wetting behavior, the same device and flow conditions can yield small, well-defined droplets. These competing effects highlight the versatility—but also the sensitivity—of flow-focusing geometries in achieving controlled droplet generation.

#### 1.1.5. Step Emulsification

Step emulsification is a passive droplet generation mechanism that relies on capillary pressure differences induced by a geometric discontinuity—typically a sudden expansion in channel depth—to drive the spontaneous breakup of droplets [71]. In contrast to the shear-based methods such as T-junctions or flow-focusing systems, step emulsification does not require high flow rates or lateral compression from a continuous phase. Instead, droplet formation is governed primarily by interfacial tension and the geometry of the microchannel.

The physical basis of step emulsification is well described by a quasi-static model, in which the interface of the dispersed phase evolves slowly enough that the system remains in near-equilibrium during most of the droplet formation process [80]. According to this model, as the dispersed phase thread exits the shallow channel and enters the deeper reservoir, a bulb begins to form downstream of the step. The mean curvature of the interface decreases as the bulb grows, requiring a corresponding adjustment in the curvature of the upstream thread to maintain pressure balance across the interface, as dictated by the Young–Laplace equation:

(2) $$\gamma \kappa =p_{i}-p_{o}$$

where γ is the interfacial tension between the inner (dispersed) and outer (continuous) phases, κ the mean curvature of the interface—defined as the sum of the principal curvatures along two orthogonal directions, $p_{i}$ is the pressure within the inner phase, and $p_{o}$ is the pressure exerted by the outer phase.

When the upstream thread can no longer compensate for the decreasing curvature of the bulb, a necking region forms at the transition. As the neck narrows, its cross-sectional diameter decreases progressively; once it approaches the nozzle height h, the critical geometric threshold, the quasi-static equilibrium breaks down, and droplet detachment is triggered by the Rayleigh–Plateau instability [81]. This mechanism, illustrated schematically in Figure 2b(i,ii), results in the spontaneous formation of monodisperse droplets with a size primarily dictated by the nozzle height, and largely independent of flow rate within the dripping regime.

This mechanism makes step emulsification particularly attractive for applications requiring high droplet monodispersity with minimal external control. Moreover, the absence of strong shear forces enhances its compatibility with fragile biological samples, such as live cells or enzymes, and facilitates integration in parallelized or portable platforms.

#### 1.1.6. Droplet Generation by Active Methods

Active droplet generators, in contrast, utilize short-duration external forces to enable on-demand droplet generation. Depending on the energy source, these methods can be classified as electrical, magnetic, thermal, or mechanical. Active systems provide highly tunable and precise droplet control but require additional external equipment, which may reduce their portability.

#### 1.1.7. Electrical Method

Electrical energy offers a versatile mechanism for controlling droplet generation in microfluidic systems, leveraging electric fields to manipulate fluid interfaces. Among the various approaches, direct current (DC) and alternating current (AC) fields have been widely explored, each presenting unique mechanisms and applications.

#### 1.1.8. DC-Controlled Droplet Generation

**Figure 3:**
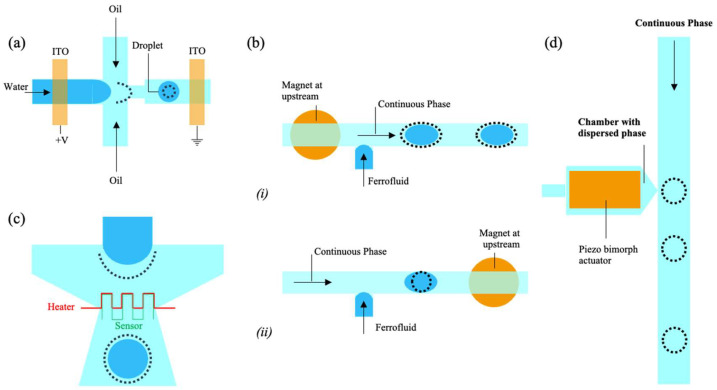
Droplet generation by active methods. (**a**) Droplet generation by applying a direct current voltage [83]. (**b**) T-junction device with ferrofluid and magnet at (**i**) the upstream position and (**ii**) the downstream position [83]. (**c**) Schematic representation of the microfluidic device designed to study the temperature dependence of the droplet formation process [83]. (**d**) Microfluidic chip for on-demand droplet dispensing: a piezoelectric actuator releases aqueous droplets into a vertical channel with immiscible fluid [83].

Link et al. pioneered the use of DC voltage in a planar flow-focusing microfluidic device (see Figure 3a) [82], where indium tin oxide (ITO) electrodes were integrated within the microchannels. In this configuration, water served as a conductive dispersed phase, while the oil stream acted as an insulating continuous phase. When a constant DC voltage was applied, charges accumulated at the water–oil interface, increasing the electric field forces. This effect led to interface deformation and the production of smaller droplets as the applied voltage increased.

The droplet generation process under DC fields can be described by the stress balance at the water–oil interface [83]:

(3) $$\gamma C=p+\left(\varepsilon_{0}E_{0,n}^{2}+\left(\varepsilon_{i}-\varepsilon_{0}\right)E_{i,n}^{2}-\varepsilon_{i}E_{s}^{2}\right)$$

where p is the internal pressure within the droplet, which balances the interfacial tension and electric stresses at the interface, γ is the interfacial tension, C is the local curvature of the droplet interface, $\varepsilon_{0}$ and $\varepsilon_{i}$ are the permittivities of the continuous (oil) and dispersed (water) phases, $E_{0,n}$ and $E_{i,n}$ are the normal components of the electric fields in the two phases, and $E_{s}$ is the tangential component of the electric field. As the voltage increases, the electric stresses grow faster than the counteracting surface tension, destabilizing the interface and resulting in smaller droplets.

Despite its effectiveness, DC-based droplet generation faces challenges such as electrode fouling and the production of charged droplets. The upstream electrode, constantly in contact with the conductive phase, can degrade over time. Moreover, the highly charged droplets may not be suitable for certain applications, such as handling sensitive biological samples. In addition, long-term operation under constant voltage can lead to the electrochemical degradation of electrode materials, compromising device durability and requiring frequent maintenance or replacement.

#### 1.1.9. AC-Controlled Droplet Generation

In contrast to DC systems, alternating current (AC) fields offer an alternative means to control droplet formation, with additional flexibility through frequency modulation. Depending on the frequency of the applied AC voltage, two regimes can be identified: low-frequency and high-frequency control.

At low frequencies, where the AC frequency is lower than the droplet generation rate, droplets form asynchronously with the applied voltage. This can lead to variations in the charge carried by individual droplets, resulting in limited uniformity.

Kim et al. demonstrated that low-frequency AC fields can stabilize Taylor cone formation for droplet generation [84]. In their setup, pulses of AC voltage (e.g., 200 ms duration) with amplitudes up to 2000 V were applied to produce droplets from the tip of the Taylor cone. However, the Taylor cone could only remain stable for limited durations due to pulsation from syringe pumps operating at low flow rates.

At high frequencies, where the AC frequency exceeds the droplet generation rate, the applied field primarily influences the wetting dynamics at the interface. This regime allows finer control over droplet size and improves uniformity. The electrowetting-on-dielectric (EWOD) effect is often leveraged in this context, where the electric field reduces the contact angle between the conductive dispersed phase and the channel surface, facilitating droplet detachment [85]. This approach has proven particularly useful in applications requiring precise droplet placement and uniformity. Nonetheless, the need for dielectric coatings and precise frequency control can complicate integration into portable or disposable devices, especially for point-of-care diagnostics.

#### 1.1.10. Magnetic Method

Magnetic fields provide a novel approach to control droplet generation, especially with ferrofluids. Yan et al. demonstrated this concept using a microfluidic flow-focusing device equipped with a magnetic tweezer [86]. By varying the magnetic field strength, the size of ferrofluid droplets could be precisely tuned. For fixed flow rates (continuous phase: 1 mL/h, dispersed phase: 0.2 mL/h), the average droplet diameter was reduced from 135 μm to 95 μm as the magnetic field increased from 0 to 60 mT. This control mechanism relies on two key effects: the magneto viscous effect, which alters the ferrofluid’s viscosity under the influence of a magnetic field, and the magnetic drag effect, where the field exerts a force that impacts droplet breakup dynamics.

Furthermore, by applying a square-wave magnetic field, droplets with alternating sizes could be periodically generated. This innovative method demonstrates the potential of magnetic manipulation for precisely controlling droplet size and distribution in microfluidic systems.

Tan et al. also investigated the formation and manipulation of ferrofluid droplets at a microfluidic T-junction under a permanent magnetic field [87] (Figure 3b). A small circular neodymium iron boron (NdFeB) magnet (3 mm diameter, 2 mm thick) was positioned beneath the device to influence droplet size. In the absence of a magnetic field, droplet size decreased linearly with an increasing flow rate of the continuous phase. When the magnetic field was applied, the droplet size was affected by the field strength, gradient, and ferrofluid magnetization. The induced magnetic force altered the droplet formation process, modifying droplet size based on magnet position: upstream placement (Figure 3b(i)) delayed formation, while downstream placement (Figure 3b(ii)) accelerated it. This effect diminished at higher flow rates due to increasing pressure and viscous forces.

While these studies demonstrate the effectiveness of magnetic fields in controlling droplet size, certain limitations must be considered. The dependence on magnetic materials restricts the range of applicable fluids, and the experimental setups can be complex. Additionally, variations in fluid properties under magnetic influence may introduce inconsistencies. Despite these challenges, the ability to dynamically adjust droplet characteristics through external magnetic fields represents a significant advancement, particularly for biomedical applications such as drug delivery and diagnostics, where precision, repeatability, and control are paramount.

#### 1.1.11. Thermal Method

Thermal methods enable the precise control of droplet generation by exploiting the temperature dependence of fluid properties like viscosity and interfacial tension. Two primary approaches are resistive heating and localized laser heating.

Resistive heating uses an integrated microheater and temperature sensor at the droplet formation site (see Figure 3c). By increasing the temperature, the viscosity and interfacial tension decrease, altering the capillary number (Ca) and allowing control over droplet size. For instance, raising the temperature from 25 °C to 70 °C can double droplet diameters, though this depends on the fluid system [88].

Localized laser heating, as shown by Baroud et al., provides high precision by focusing a laser at the droplet site, inducing the Marangoni effect and surface tension gradients [89]. This method can double droplet size and supports advanced manipulations like merging and splitting. Pulsed lasers enable rapid droplet generation at rates up to 10,000 droplets per second, though the careful regulation of system temperature is essential to maintain stability.

Both methods provide flexible thermal control for droplet generation, enabling the precise modulation of droplet size and formation dynamics. However, they also present certain limitations. Resistive heating requires integrated microheaters and sensors, adding complexity to device fabrication and operation, while localized laser heating demands precise alignment and power regulation to avoid unwanted thermal effects. Additionally, thermal techniques may cause unintended changes in fluid properties, potentially affecting droplet stability and reproducibility. Despite these challenges, they remain valuable for applications ranging from lab-on-a-chip systems to high-speed microfluidics, particularly where tunable and rapid droplet manipulation is required.

#### 1.1.12. Piezoelectricity Control

Piezoelectric actuation offers rapid and precise droplet generation by either dispensing droplets on demand or modulating the droplet formation process.

In dispensing applications, such as those demonstrated by Xu et al., a piezoelectric pulse overcomes interfacial tension to inject droplets into the continuous phase [90]. The droplet size is controlled by adjusting the pulse duration and voltage, allowing for production rates of up to 5 kHz and pulse widths as short as 200 μs. This is over 200 times faster than pneumatic methods (see Figure 3d). Adjustments in pulse patterns allow for varied droplet volumes or double droplets per pulse.

Flow-focusing configurations, as used by Bransky et al., improve efficiency by reducing interface deformation, minimizing satellite droplets and enhancing control through precise liquid height adjustments [91].

Piezoelectric systems combine speed, precision, and adaptability, making them ideal for advanced microfluidic applications. This methodology is well-established in industrial settings, particularly in ink-jet printing, where precise droplet control is critical for print quality (see, for example, studies on waveform optimization to minimize residual vibrations and crosstalk in multidrop ejection methods [92]). Given this proven scalability and the ability to fine-tune droplet behavior, piezoelectric systems are a promising candidate for industrial-scale microfluidic applications. However, the mechanical stress induced by continuous actuation may lead to material fatigue, especially in polymer-based microchips. Furthermore, the alignment and integration of piezoelectric actuators with microfluidic networks can increase fabrication complexity and overall system cost.

#### 1.1.13. Comparative Assessment of Droplet Generation Techniques

**Figure 4:**
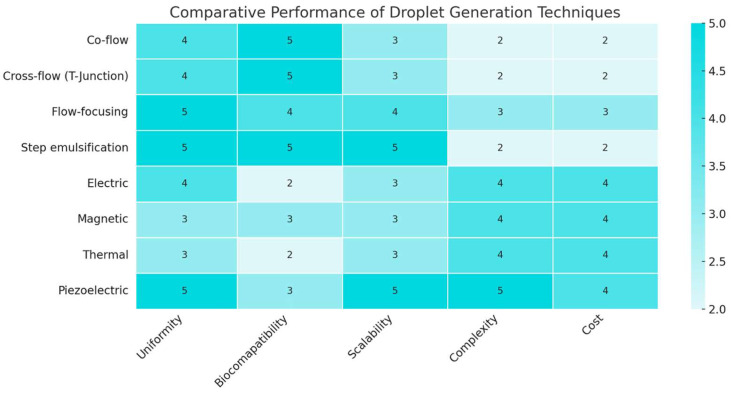
Comparative heatmap of droplet generation techniques based on five performance categories: droplet uniformity, biocompatibility, scalability/throughput, system complexity, and cost. Passive methods (co-flow, cross-flow/T-junction, flow-focusing, and step emulsification) and active methods (electric, magnetic, thermal, and piezoelectric) are scored qualitatively on a scale from one (poor) to five (excellent) based on literature-reported capabilities and implementation requirements. This graphical overview highlights the strengths and trade-offs of each approach, supporting selection based on specific application needs.

Figure 4 summarizes the performance of key droplet generation strategies, categorized as passive (co-flow, T-junction, flow-focusing, and step emulsification) or active (electric, magnetic, thermal, and piezoelectric). These were evaluated across five core metrics: droplet uniformity, biocompatibility, scalability, complexity, and cost.

Passive methods stand out for simplicity, affordability, and high biocompatibility. Among them, step emulsification and flow-focusing are particularly effective for generating monodisperse droplets, especially in low-flow or parallelized setups. Their main limitation lies in limited tunability and strong dependence on channel geometry and wetting conditions.

Active methods offer precise, on-demand control, with piezoelectric and electric actuation enabling high uniformity and scalability—already leveraged in sectors like inkjet printing. However, they often involve greater complexity and lower biocompatibility. Magnetic and thermal methods provide niche control mechanisms but require specialized materials or external actuation systems.

The optimal strategy depends not only on technical performance (as shown in Figure 4), but also on intended use. Passive techniques are ideal for resource-limited or biologically sensitive contexts. In contrast, active systems better serve programmable and high-throughput workflows where control and adaptability are essential.

For successful industrial translation, microfluidic platforms must evolve toward integrated, scalable, and user-friendly systems. This involves minimizing reliance on bulky peripherals, enabling automation, and standardizing designs—crucial steps toward adoption in diagnostics, drug screening, and biomanufacturing.

## 2. Droplet Trapping and Manipulation Techniques

In the realm of contemporary microfluidics, the methodologies of trapping, selecting, and the on-demand release of droplets have attained fundamental processes, exhibiting a wide spectrum of applications ranging from biomedical research to chemical analysis and drug screening. The capacity to meticulously manipulate microspheres or droplets enables dynamic control over their position and content, thereby facilitating in situ studies such as chemical reactions, cell cultures, and time-lapse analyses. For instance, on-demand trapping is critical for experiments where varying the concentration of reagents, such as inhibitors, in individual droplets is essential for high-precision drug screening analysis.

Among trapping techniques, passive methods exploit fluid dynamics phenomena, such as hydrodynamic resistance, to direct droplets toward paths of lower resistance or trap them in geometrically defined wells or traps [93,94]. While these methods are straightforward to implement, the precise control of trapping can depend on parameters such as droplet size and the spacing between droplets travelling along the main channels. For example, microfluidic devices based on hydrodynamic resistance have been designed to trap individual droplets in a controlled manner, although they often require variable pressure sources to activate or deactivate trapping, adding some complexity to the system [95].

In contrast, active methods employ physical phenomena such as electric, magnetic, or acoustic fields to manipulate droplets with greater precision and flexibility. These approaches include the use of embedded electrodes to generate electric fields (e.g., DC or AC fields) [96], elastomeric valves for on-chip control [52], or external actuators such as surface acoustic waves [97] and laser heating [98]. While these methods can provide more sophisticated control, the use of external fields or non-biocompatible materials can introduce operational complexities or risks to the integrity of biomolecules.

The subsequent section will provide a comprehensive exploration of the predominant on-demand trapping techniques, which are classified into two overarching categories: passive methods, founded on mechanical and fluid dynamic phenomena, and active methods, which leverage interactions with external physical fields. An overview of key approaches and representative devices will be presented, highlighting the advantages, limitations, and specific applications of each category.

### 2.1. Passive On-Demand Trapping Techniques

Passive droplet trapping refers to a class of microfluidic strategies that enable the immobilization and spatial control of droplets without the use of external fields. This approach relies entirely on intrinsic physical phenomena—such as fluid dynamics, pressure gradients, and geometric constraints—to manipulate droplets within microchannels.

These techniques are particularly appreciated for their simplicity of implementation and compatibility with compact, low-cost systems. They leverage variations in hydrodynamic resistance or channel geometry to guide droplets into designated traps or wells, ensuring precise positioning. Importantly, passive methods operate without the need for actuators, electrodes, or external energy sources, which makes them especially attractive for applications requiring scalability, portability, or minimal instrumentation.

#### Hydrodynamic Trapping Techniques

A notable passive technique is hydrodynamic trapping, which employs variations in hydrodynamic resistance within microchannels to direct droplets towards paths of reduced resistance or to immobilize them in predefined positions. These systems offer effective control, although their precision can be influenced by droplet size, the spacing between droplets, and channel geometry.

A number of studies have been published in this area, including several technical articles that propose platforms for the dynamic trapping of droplets obtained from water-in-oil (W/O) emulsions. However, it should be noted that the majority of these studies focus on the trapping process itself rather than on its direct application in the biological field. Consequently, there is a need to explore the potential use of these platforms in biological processes involving encapsulated cells, such as cell culture in three-dimensional (3D) microenvironments or single-cell analysis. Such applications could include advanced techniques such as mass spectroscopy [99] or electrochemical analysis [100], opening new perspectives for the integration of these technologies in the fields of biology and biotechnology.

A passive droplet trapping strategy in microfluidics involves exploiting the balance between net Laplace pressure and hydrostatic pressure to achieve stable immobilization of the droplet [101]. As demonstrated by Simon et al. [101], this mechanism relies on the pressure difference across the front and rear interfaces of the droplet, induced by channel geometry—particularly a narrowing of the microchannel—which creates a curvature gradient. This pressure difference, commonly referred to as Laplace pressure, can be described by the following equation:

(4) $$∆p_{L}=\gamma \left(\frac{1}{R_{y,f}}-\frac{1}{R_{y,b}}\right)$$

where γ is the surface tension of the fluid, while $R_{y,f}$ and $R_{y,b}$ are the curvature radii of the drop in the front and rear sections, respectively. The resulting pressure acts as a restoring force that resists the motion of the droplet, enabling passive trapping without the need for active control mechanisms.

Conversely, the hydrostatic pressure, exerted by the continuous fluid flowing in the channel, acts to propel the drop forward. In its general form, the Hagen–Poiseuille law provides a simplified estimation of the pressure drop associated with viscous flow in confined geometries:

(5) $$∆p_{H}=\frac{8\mu LQ}{\pi R^{4}}$$

where μ denotes the viscosity of the fluid, L is the length of the channel, Q is the volumetric flow rate, and R is the effective hydraulic radius of the channel. While this formulation strictly applies to cylindrical geometries, it can serve as a reasonable approximation in more complex microfluidic designs.

**Figure 5:**
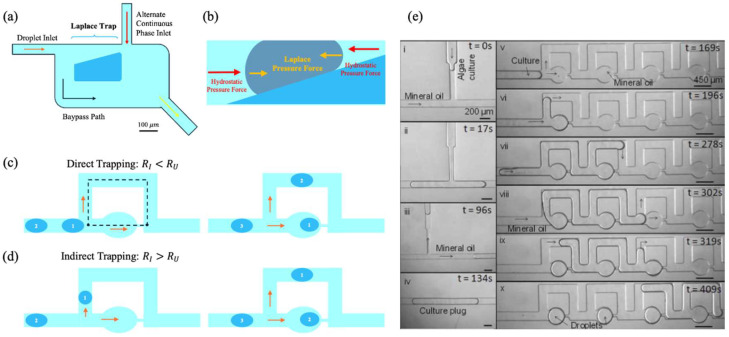
Representative images of the trapping process using pressure variations and bypass channels. (**a**) Major features of the Laplace trap are illustrated. (**b**) Force balance showing the Laplace pressure forces (yellow) at the droplet interfaces and hydrostatic pressure forces (red) from the continuous phase in the microfluidic channels. Arrow size indicates force magnitude [102]. (**c**) Droplet capture in the traps using the direct trapping approach, where R_l_ < R_u_. (**d**) Droplet capture in the traps using the indirect trapping approach, where R_l_ > R_u_ [103]. (**e**) Time-stamped images showing the formation of a single algae culture plug (**i**–**iv**) and droplet arrays (**v**–**x**). Figure (**e**) reprinted with permission from Ref. [103], copyright 2012 by John Wiley and Sons.

When the Laplace pressure, resulting from the curvature-induced pressure gradient across the droplet interface, balances the hydrostatic pressure applied by the continuous phase, the droplet decelerates and eventually comes to rest. This equilibrium condition—$∆p_{L}=∆p_{H}$—forms the fundamental operating principle of Laplace traps, as illustrated in Figure 5a.

Figure 5b illustrates the balance of forces, showing the direction of the Laplace pressure forces (in yellow) acting on the drop interfaces, both front and rear, and the hydrostatic pressure forces (in red) provided by the continuous fluid flowing in the channel. Arrows of different sizes indicate the relative intensity of each force.

Using MATLAB simulations and three-dimensional models using CFD software, CFD-ACE, (ESI Group, Huntsville, AL), Simon et al. demonstrated that various physical parameters, such as droplet size and channel geometry, influence the balance of forces. For example, it was observed that the size of the channel narrowing directly affects the Laplace pressure: narrower channels generate higher pressures, thus increasing the probability of the droplet being trapped. However, if the constriction is too small, it may be difficult to release the drop later, necessitating a fine balance between drop size and channel openings.

Simulations also highlighted the importance of surface tension, γ, which varies depending on the chemical composition of the system. For example, water droplets in oils with high surface tensions, such as light mineral oil, develop higher Laplace pressures, stopping more easily. Conversely, the use of surfactants, which reduce surface tension, can prevent trapping, requiring changes in channel geometry to balance this effect.

Despite significant theoretical and experimental advancements, a key challenge of this approach is the lack of an intuitive method to determine the optimal parameters for trapping. The necessity to define the channel dimensions, droplet sizes, fluid flow rate and other parameters in advance renders the approach rather complex. This limitation restricts the system’s adaptability and necessitates preliminary optimization, a process that may not always be practicable, particularly in contexts involving variability or complexity. In these systems, the hydrodynamic resistance of the network and that of the individual traps determine the movement and immobilization of the droplets.

In their analysis of microfluidic manipulation strategies, Bithi et al. [102] identified two main modes for trapping droplets in the microfluidic network—referred to by the authors as *direct* trapping (Figure 5c) and *indirect* trapping (Figure 5d)—both of which are based on changes in the hydrodynamic resistance within the network.

In the process of *direct* trapping, the initial droplet enters the lower branch of a microfluidic loop, which contains a hydrodynamic trap. This phenomenon occurs because the hydrodynamic resistance of the lower branch ($R_{l}$) is lower than that of the upper branch ($R_{u}$). Following the initial trapping event, the overall resistance of the lower branch is increased, which in turn forces the subsequent droplet to enter the upper branch. This process serves to prevent further entry into the same trap and is illustrated in Figure 5c.

In the configuration of *indirect* trapping, the hydrodynamic resistance of the lower branch ($R_{l}$) is greater than that of the upper branch ($R_{u}$). Consequently, the initial droplet is deflected towards the upper branch. This results in a temporary increase in the hydrodynamic resistance of the upper branch, thereby obstructing the flow in that direction. Consequently, the second droplet is directed towards the lower branch, where it becomes ensnared within the hydrodynamic trap (see Figure 5d).

The microfluidic device developed by Dewan et al. is based on these principles for the long-term storage of nanolithium droplets [103]. In a similar manner, the microfluidic network designed by the same authors uses hydrodynamic traps to immobilize droplets in predetermined positions (see Figure 5e), thereby maintaining stable volumes through the precise control of water permeation. Trapping efficiency was optimized by carefully configuring the hydrodynamic resistance of the network in relation to that of the droplets, as described in the work of Bithi et al.

**Figure 6:**
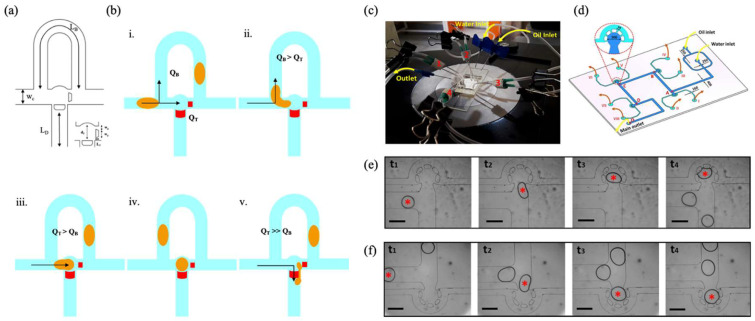
Illustrative images of the trapping process through variations in hydrodynamic resistance. (**a**) Microchannel dimensions for the trapping design. (**b**) Droplet trapping scenarios: (**i**) Flow rates Q_B_ and Q_T_. (**ii**) Droplet enters the bypass channel instead of the trap. (**iii**,**iv**) Droplet enters the trap. (**v**) Droplet enters the trap and pushes into the diluting stream. (**c**) Experimental setup. Four pairs of binder clips are attached to the PVC tubes for closing and opening the side channels connected to the traps. (**d**) Schematic of a microfluidic device enabling on-demand droplet trapping via hydrodynamic resistance modulation. Droplets, generated at a flow-focusing junction, travel through the main channel and are diverted into side traps when the associated binder clip is opened, lowering the resistance of the corresponding side path. Once a droplet enters a trap, closing the clip immobilizes it. To ensure effective trapping, the main channel’s resistance is increased by adding an oil-filled outlet pipe, favoring flow into distal traps. (**e**,**f**) Sequential images of on-demand droplet trapping in two different traps of the microfluidic device. Entrapped droplets are indicated by red stars. Figure (**c**–**f**) reprinted with permission from Ref. [105]. Copyright 2024 by American Chemical Society.

These studies laid the foundation for the work of Courtney et al., who further improved the trapping devices by introducing mechanisms for the controlled release of droplets [104]. Through a design that includes circular traps and bypass channels (see Figure 6a), Courtney et al.’s system allows direct control over the release of droplets, improving flexibility and reducing the time required for screening operations.

This innovative approach addresses some of the limitations of traditional methods, such as high reagent consumption and long reaction times associated with 96-well plate-based systems. In their device, trapping is achieved by exploiting variations in hydrodynamic resistance and pressure in microchannels, designed with a complex network of circular traps and bypass channels.

The fundamental mechanism of the trapping system is predicated on the modulation of the relationship between the hydrodynamic flow through the traps and the flow through the bypass. In circumstances where the flow towards the trap exceeds that of the bypass, droplets are trapped at the traps ($Q_{T}>Q_{B}$, see Figure 6b(i–v)). Conversely, by adjusting the pressure in the release channels, the droplets can be released with precision. This design offers a stable droplet trapping mechanism that does not necessitate active techniques such as electrodes or laser heating, enhancing efficiency and reducing system complexity.

The efficacy of the method has been demonstrated in a drug screening application for the inhibition of tau peptide aggregation, a phenomenon related to Alzheimer’s disease. The system’s precision enables the generation, mixing, and trapping of droplets within seconds of their formation, facilitating rapid and repeatable analysis. This strategy signifies a substantial advancement over conventional methods, as it curtails sample consumption by approximately 200-fold and reaction time to a mere 2.5 min, while maintaining the robustness and reliability of measurements.

Nevertheless, despite its advantages, the system has certain limitations. The design of channels necessitates meticulous precision to calibrate the equilibrium between flows traversing traps and bypasses. These parameters appear to be influenced by factors such as channel dimensions, fluid viscosity, and applied pressures.

An innovative approach worthy of attention in the field of on-demand droplet trapping is the one developed by Besanjideh et al., which presents a highly flexible and easily implemented methodology [105]. The proposed system is distinguished by the utilization of rudimentary tools, such as flexible PVC pipes and binder clips (see Figure 6c), which enable the hydrodynamic resistance to be modulated without the necessity for built-in valves or sophisticated actuators. This feature greatly simplifies the implementation and allows water-in-oil droplets to be selected and trapped in predefined traps within a microfluidic device.

The device design is based on a precise control of the hydrodynamic resistances in the main and side channels (see Figure 6d). Droplets, initially generated in a flow-focusing joint, are transferred into the main channel where they can be directed towards traps strategically placed along the route. These traps are connected to side channels whose hydrodynamic resistance can be modulated, allowing the flow to be selectively diverted to the desired trap. The opening and closing of the side channels regulate the path of the droplets, allowing highly selective trapping.

For effective trapping, it is crucial that the hydrodynamic resistance of the side channels connected to the traps is lower than that of the main channel. However, the trapping of droplets in the final traps can be problematic, as the hydrodynamic resistances of the paths tend to equilibrate. To overcome this difficulty, Besanjideh et al. propose to increase the hydrodynamic resistance of the main channel by adding an oil-filled pipe to the outlet, thus creating a significant difference in resistance that facilitates trapping in the end traps as well.

The on-demand trapping process is developed in several stages, combining precise hydrodynamic control and the use of clips to modulate flows within the device. Initially, mineral oil is injected into the device through a syringe pump until oil is observed to rise in the PVC pipes connected to the side channels. Subsequently, four pairs of clips are applied to the PVC pipes to block the flow in the side channels, allowing the oil to flow exclusively into the main channel for approximately 20 min, without water. This step increases the hydrodynamic resistance of the main path, balancing the pressure within the device.

Once the pressure is balanced, distilled water is injected at a controlled flow rate of 0,1 μL/min, forming aqueous droplets at the flow-focusing junction. During this phase, the clips remain closed, increasing the hydrodynamic resistance of the side channels to theoretically infinite values, thus preventing the droplets from diverting towards the traps and keeping them in the main channel.

To selectively trap a single droplet, the clip pair associated with the desired trap is opened. This instantly reduces the hydrodynamic resistance in the side channels, making them preferential for flow and diverting the droplet towards the trap. Once the droplet reaches the trap, the clips are closed again, immobilizing the droplet through the presence of micropillars. This approach enables the precise and highly selective trapping of droplets, as demonstrated by sequential images illustrating the trapping process for droplets between 300 and 350 μm in size (Figure 6e,f).

The method proposed by Besanjideh et al. offers numerous advantages, including the simplicity of implementation and the possibility of using it as a platform for individual microreactors. The traps can accommodate reagents and the geometry of the side channels allows two-way, independent flow control.

Despite the efficiency and flexibility of the system, successful trapping depends on the precise synchronization of the opening and closing of the clips, requiring slow flows and carefully controlled droplet sizes. The use of CFD simulations made it possible to optimize the geometric and flow parameters, reducing manufacturing costs and improving the overall performance of the system.

In conclusion, Besanjideh et al.’s method represents a significant step towards a practical and scalable solution for on-demand trapping, paving the way for a wide range of applications, ranging from small-scale biological tests to the creation of modular platforms for droplet handling in microfluidic systems.

### 2.2. Microfabricated Structures

Droplet trapping is a crucial aspect in many microfluidic applications, especially in contexts where the precise control of droplet position is required for long-term experiments or in situ analysis. Microfabricated structures, such as microwells, ‘U-shaped’ structures and bilayer wells, are examples of passive techniques that exploit spatial confinement to precisely manipulate and control droplets. These techniques allow for well-defined environments for the temporal and spatial monitoring and analysis of biological or chemical processes, which is crucial for complex studies such as drug response or the formation of cellular aggregates.

#### ‘U’ Shaped-Entrapping

**Figure 7:**
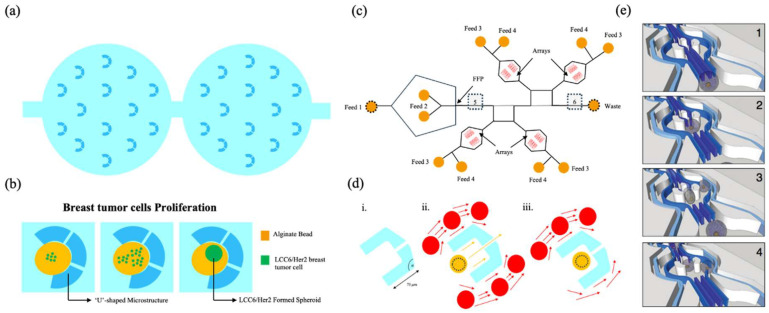
Representative images of the trapping process using ‘U’-shaped microstructures. (**a**) Alginate beads trapped in the micro sieves of the microfluidic chip, with each bead containing approximately 100 cells at the time of loading. (**b**) Schematic representation of LCC6/Her2 breast tumor cells proliferating and forming multicellular spheroids while encapsulated in alginate beads [106]. (**c**) Schematic of a microfluidic device with four trap arrays for droplet generation, trapping, incubation, monitoring, and release. Droplets form at the flow-focusing point (FFP), where an aqueous stream (Feed 2) intersects with two oil streams (Feed 1). Once a uniform droplet size is reached, withdrawing oil via Feed 3 directs stable droplets into the trap arrays. Prior to stabilization, keeping Feed 3 closed or reversing its flow allows polydisperse droplets to bypass the traps and reach the waste outlet. Trapped droplets can be monitored via bright-field or fluorescence imaging, with laser-induced fluorescence measurements performed at designated points (grey boxes). Droplets are released by injecting oil through Feed 4. (**d**) Droplet trapping arrays: (**i**) Design of an individual trap with a 110° angle (α) at the rear, improving droplet retrieval to over 90%. (**ii**) Schematic flow profile of droplets approaching the trap. (**iii**) Flow profile when a droplet is trapped; additional droplets bypass the occupied trap, ensuring single-droplet capture [107]. (**e**) Illustration of the trapping mechanism in a series of time-resolved sketches. Faster flow is observed in the middle of the channel (dark blue) and slower flow by the channel walls (light blue). Figure (**e**) reprinted with permission from Ref. [108], copyright 2019 by John Wiley and Sons.

The first study we review here is that of Yu et al., who propose a microfluidic droplet system for the creation of multicellular tumor spheroids, used as a model for drug testing [106]. In this work, the process of trapping droplets takes place within a microfluidic system that utilizes ‘U-shaped’ structures to hold alginate beads containing tumor cells in place. The droplets, initially formed in a droplet-generating chip, are composed of a suspension of alginate and tumor cells; once gelled, they are trapped in microsieves in the microfluidic chip (see Figure 7a). This system enables the maintenance of each bead in a fixed position during the entirety of the process, encompassing cell proliferation, spheroid formation, drug treatment, and imaging. The use of microsieves ensures the spatial stability of the beads, allowing the precise and continuous monitoring of the drug response, as illustrated by the greater resistance of the spheroids compared to monolayer cultures in the case of doxorubicin. Passive trapping in these structures thus enables well-defined environments for long-term cultures and in situ analyses (see Figure 7b).

In the work of Huebner et al. [107], the droplet trapping process is also based on the use of ‘U’-shaped traps, similar to the system described above by Yu et al. This study employs a single-layer microfluidic PDMS device to efficiently trap and release picoliter droplets in an array of ‘U’-shaped traps. Droplet formation is achieved through a flow-focusing geometry, where an aqueous stream interacts with two oil streams, enabling the generation of monodisperse droplets. Once a uniform droplet size is reached, reversing the oil flow (via Feed 4) facilitates the trapping of droplets within each individual trap. The schematic design of this device is illustrated in Figure 7c.

Similarly to Yu et al.’s system, the ‘U’ trap configuration prevents multiple droplets from occupying the same trap, allowing unique trapping for each one. Upon the entry of a droplet into a trap, it obstructs the oil flow through the drain, thereby preventing the entry of other droplets, as illustrated in Figure 7d. This capacity to isolate each droplet is crucial for the characterization of biological or chemical reactions within a single compartment, as demonstrated by Huebner et al. in their study of phenomena such as cell aggregation and enzymatic reactions.

Another important similarity with the work of Yu et al. is the possibility of releasing trapped droplets for further analysis. In Huebner et al., the release of the droplets occurs by means of a flow of oil that reverses direction, releasing the droplets from the traps. Furthermore, unlike Yu et al., who focuses on encapsulating tumor cells, Huebner et al. used the device to monitor the response of droplets to enzymatic reactions, but both works share the basic principle of ‘U’ traps for the controlled trapping and release of droplets.

In a similar manner, the droplet trapping process proposed by Kleine-Brüggeney et al. employs ‘U-shaped’ traps that are undersized in relation to the diameter of the droplets themselves [108]. In this configuration, the droplets, with an approximate diameter of 80 µm, are directed into the traps by means of a mechanism that operates on the principles of hydraulic pressure and the kinetic energy generated by the liquid flow. The beads, composed of hydrogel, are initially positioned in front of the trap entrance; however, it is only when the flow reaches a certain speed that the pressure exerted compresses them, forcing them to enter the trapping site. An illustrative sequence of the trapping process is presented in Figure 7e.

This methodology contrasts with alternative approaches that rely on a flow passing through a bypass channel to achieve trapping. In this configuration, the primary mechanism of trapping is determined by the interplay between the kinetic energy of the beads and the hydraulic pressure field generated by the flow. At low flows, the beads stop before entering the trap, while at higher flows, the hydraulic pressure generated is sufficient to deform the beads and force them into the traps. This method utilizes the deformability of the beads to achieve precise trapping without the use of bypasses or other auxiliary structures.

The beads, once trapped, are precisely arranged in the device, allowing an accurate spatial immobilization of the cells they contain. This approach was used to study the long-term behavior of murine stem cells in a three-dimensional environment by monitoring their differentiation and clonal behavior through advanced real-time confocal microscopy techniques.

### 2.3. Rails and Anchors—A Different Trapping Technique

In light of the earlier concept of flow-induced hydraulic thrust in continuous phase, the focus now shifts to works that leverage this physical principle, with a particular emphasis on those employing microfluidic devices with variable internal depths. These devices are designed to create rails or guide lines, as well as anchor points for droplets, allowing the precise control of their movement and behavior. Such systems are based on the variation in the spatial confinement of the droplets, a phenomenon that generates surface energy gradients capable of influencing the position and stability of the moving droplets within the channel.

The work of Dangla et al. makes a significant contribution to the study of the interaction between fluid droplets and microstructures in microfluidic channels, providing a solid theoretical basis for the precise control of their behavior [109]. In microfluidics, the ability to anchor and manipulate droplets is essential for numerous applications, including chemical analysis, synthetic biology, and the production of micrometer-scale materials. The study focuses on how geometric variations, such as microcavities or protuberances engraved on the channel surface, can generate surface energy gradients capable of stabilizing droplets at predetermined points or guiding them along specific paths.

**Figure 8:**
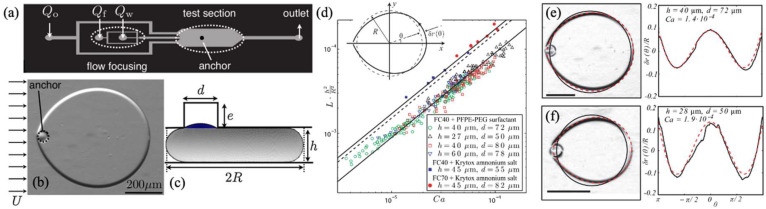
Rails and anchors. (**a**) Schematic of a microchannel that consists of a flow focuser to generate water drops in oil and of a test section with a single anchor. (**b**) A top-down image of a droplet held in place by an anchor against a mean external flow U. (**c**) Surface Evolver rendering of an anchored droplet of outer radius R inside the microchannel of height h over an anchor of diameter d and of depth e. (**d**) Sketch of the polar coordinates for the deformations δr(θ) from the static radius R. (**e**,**f**) Left: Images of anchored droplets deformed by the oil flow. The dashed lines show the prediction from Equation (11), while the solid line shows the equivalent circle of radius R. Right: Comparison between the deformations δr(θ)/R extracted from the microscope images on the left (solid line) and the theoretical curve (dashed line) obtained from Equation (11), using nine as a pre-factor. Figure (**a**–**f**) reprinted with permission from ref [109], copyright 2011 by American Physical Society.

Through a combined theoretical and experimental approach, the authors analyze how cylindrical anchors embedded in PDMS microchannels create zones of reduced confinement that serve as surface energy sinks for the droplets (Figure 8a).

When a static droplet interacts with a microfluidic anchor—typically a microcavity or geometric irregularity—it partially or completely penetrates the anchor, assuming a spherical cap configuration. The extent of penetration depends on the geometric ratio b=dh, where d represents the diameter of the anchor and h the height of the microchannel through which the drop passes (see Figure 8b,c). As *b* varies, the behavior of the droplet also varies. For b > 2, the droplet fills the anchor entirely, while for *b* ≤ 2, the droplet only partially enters, resulting in a stable canopy.

This interaction leads to a reduction in surface area ∆A, and hence in surface energy:

(6) $$∆E_{\gamma }=\gamma ∆A\;$$

The change in area is captured by a dimensionless function S(b), which depends on anchor geometry. As long as $S\left(b\right)>0$, the surface energy decreases, promoting trapping. The corresponding anchoring force can be estimated by the following:

(7) $$F_{\gamma }^{*}\propto \frac{\left|∆E_{\gamma }\right|}{d}\approx \gamma \frac{\pi }{2}hS\left(b\right)$$

In the presence of an external flow, the drag force $F_{d}$ acting on the droplet tends to displace it. The system is approximated as a Hele–Shaw cell [110], where the channel height h is much smaller than its in-plane dimensions. Under this approximation, the flow is quasi-two-dimensional and the velocity field averaged over height satisfies Darcy’s law:

(8) $$\vec{\nabla p}=-\frac{12\mu }{h^{2}}\vec{U}$$

Here, μ is the dynamic viscosity and $\vec{U}$ is the in-plane velocity field. In this regime, viscous shear plays a secondary role, and the dominant contribution to the drag arises from pressure differences across the droplet interface.

Asymptotic studies of pressure distribution around inviscid droplets in Hele–Shaw flow [111,112] provide an expression for the outer pressure field $p_{o}(x)$ acting on the droplet:

(9) $$p_{o}(x)=-24\frac{\mu U}{h^{2}}x+Cst$$

By integrating this pressure over the droplet interface, the total drag force is given by the following:

(10) $$\vec{F_{d}}=24\pi \frac{\mu UR^{2}}{h}$$

where R is the in-plane radius of the droplet and U is the average velocity of the external flow. This drag force, resulting from the external pressure field, counteracts the stabilizing effect of the anchor, which acts through a surface energy gradient. The balance between these two opposing effects determines whether the droplet remains pinned or is released by the flow.

To validate the theoretical expression for the external pressure field $p_{o}(x)$, the authors analyzed the deformation of droplets anchored in microcavities under flow. By applying the Laplace equation, which links the pressure jump across the droplet interface to its mean curvature, the authors related the droplet shape to the pressure distribution induced by the external flow.

Assuming small deviations from a circular shape, they described the interface deformation as a radial deviation δr(θ) from the unperturbed droplet radius. The resulting expression shows that the deformation amplitude depends on the capillary number Ca=μUγ:

(11) $$\frac{\delta r\left(\theta \right)}{R}=15.3Ca\frac{R^{2}}{h^{2}}\left(1-\theta sin\theta \right)$$

This relationship is experimentally validated by plotting the dimensionless deformation parameter $Lh^{2}/R^{2}$ versus Ca (Figure 8d), showing data collapse across multiple fluidic and geometric conditions. The slope variations are attributed to differences in surfactant adsorption kinetics, which influence interfacial stress distributions. By replacing the theoretical prefactor 15.3 with the slope of the experimental fits, the model accurately captures droplet shape under varying flow rates (Figure 8e,f).

The work of Dangla et al. established the basis for the control of fluid droplets in microchannels through geometric variations, thereby demonstrating the significance of surface energy gradients in anchoring and stabilizing droplets. Expanding on this, Abbyad et al. [113] introduces a complementary and equally revolutionary methodology, focusing on the use of etched patterns for finer control of droplet movement and properties. The technique employs microstructures designed to anchor the droplets in specific regions or to guide them along well-defined paths through linear, track-like grooves. This enhancement of accuracy in two-dimensional control is accompanied by the ability to modulate the chemical conditions within the droplets themselves, thereby expanding the range of potential applications.

In the same spirit of advancing droplet-based microfluidics, subsequent studies have further developed and refined these approaches, introducing novel technologies and enhancing droplet manipulation for a variety of applications. In Table 2, a summary of these key works is presented, highlighting their primary technologies, innovations, and applications.

Therefore, rails and anchors are extremely useful tools for passively guiding droplets in contexts where highly specific manipulation is crucial. For example, the study advanced by Duchamp et al. highlights the pivotal role of rails in achieving precise control over droplet sorting and co-encapsulation [114]. The microfluidic system employs a dual-rail mechanism, where droplets are sorted based on size: droplets larger than 35 µm are guided along a lower rail, while those smaller than 20 µm remain on an upper rail. This innovative use of rails ensures accurate filtering and positioning of droplets for subsequent trapping within a floating trap array (FTA). The dual-rail system is critical for achieving high-efficiency pairing of droplets, facilitating the merging process via electrocoalescence to produce co-encapsulated droplets. This rail-based approach enhances precision, supporting high-throughput creation of complex droplet libraries for advanced biochemical studies.

Building on this concept, Sart et al. extended the concept of rail-guided droplet control into the realm of three-dimensional (3D) cell culture [116]. Their research highlights the integration of geometric droplet manipulation with dynamic modulation of the cellular environment, creating a versatile platform for high-density cytometric analysis. By employing pre-defined grooves and anchors, the system guides cell-laden droplets, stabilizing them at specific anchor points. These droplets, containing cells suspended in agarose, serve as microenvironments for the formation of cellular spheroids. The solidification of agarose at low temperatures ensures droplet stability, while the replacement of oil with an aqueous medium supports long-term culture. This setup enables the formation of spheroids with a mean diameter of ~73 µm, overcoming limitations in nutrient and oxygen diffusion.

A key innovation of Sart et al.’s work is the selective retrieval of spheroids. Using a focused infrared laser, individual agarose droplets are melted locally, and the spheroids are detached and transported off-chip in a stream of culture medium. Retrieved spheroids remain viable and exhibit migratory capabilities when replanted on 2D substrates. This selective isolation of 3D cellular structures underscores the adaptability and utility of the platform for applications requiring precise control over cell culture and analysis.

Similarly, Segaliny et al. introduce selective release as a central feature of their droplet-based platform, but with a focus on single-cell applications, particularly in cancer immunotherapy [117]. Their system integrates droplet microfluidics with functional screening and real-time monitoring of TCR T cell activation. Like Sart et al., Segaliny et al. employ geometric structures to anchor droplets efficiently, enabling the tracking of individual T cell clones during their activation and interaction with target tumor cells. Crucially, the platform includes a highly specific sorting mechanism for the selective recovery of activated clones. This is achieved through laser-induced cavitation, where ultraviolet light is focused on individual droplets, precisely rupturing the targeted compartment to release the desired cells for downstream molecular analysis.

By combining precise droplet anchoring with selective release mechanisms, Segaliny et al. demonstrate the potential of droplet-based platforms to address key challenges in therapeutic screening, such as identifying rare, functional T cell clones. Together with the innovations of Sart et al., this work highlights the versatility of droplet-based systems for high-throughput, selective analysis and recovery, bridging fundamental research with translational applications in personalized medicine and immunotherapy.

### 2.4. Active On-Demand Trapping Techniques

Active trapping methods represent a powerful alternative to passive approaches, offering superior precision and adaptability by leveraging external physical fields to manipulate droplets dynamically. Unlike passive strategies, which rely solely on intrinsic fluid dynamics and device geometry, active methods utilize interactions with external forces such as electric, magnetic, or acoustic fields to achieve on-demand control over droplet positioning and retention.

These methods enable the selective trapping, release, or coalescence of droplets with exceptional accuracy, overcoming many of the limitations associated with size dependency or pressure variability inherent to passive techniques. As a result, active trapping systems have found widespread applications in advanced microfluidic platforms, particularly in scenarios requiring high-throughput analysis, single-cell studies or complex biochemical assays. Some of the most promising and innovative works in this area, showcasing a variety of active trapping techniques and their diverse applications, are summarized in Table 3. These studies highlight the versatility and efficiency of active methods in a wide range of experimental settings.

Active methods offer the significant advantage of being integrated with passive devices, thus combining the use of external forces with the intrinsic characteristics of fluid dynamics. This synergy enables the exploitation of both natural fluid phenomena and interactions induced by external sources, ensuring highly specific and high-throughput droplet manipulation.

**Figure 9:**
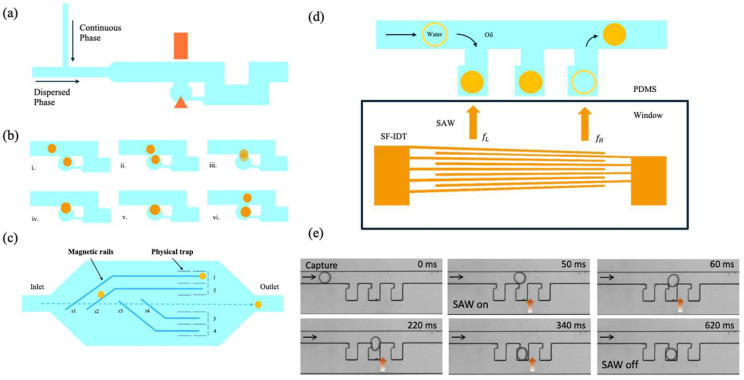
Representative images of trapping using active techniques. (**a**) Schematic drawing of the microfluidic platform showing the detailed structure of the channels, microwell, and electrodes; shaded areas represent electrodes embedded in the platform. (**b**) Trapped droplet is fused with a droplet passing by (**i**–**iii**); fused droplet gets trapped in the microwell again (**iv**–**vi**); and after removing the electric field, no more fusion occurs (6) [96]. (**c**) Schematic of a microfluidic device combining magnetic rails (blue) and physical traps (gray). Magnetic droplets (orange) follow the main flow unless deviated by localized fields. When the magnetic field is activated, droplets are guided along the rails into traps, which retain them even after the field is turned off. The system enables precise, on-demand droplet routing, trapping, release, and merging. [122]. (**d**) Schematic of the acousto-microfluidic droplet capture and release device, featuring a PDMS channel and a piezoelectric substrate with slanted finger interdigitated transducers (SF-IDT). Droplets are captured or released using acoustic radiation force (ARF), with the SAW beam position adjusted by tuning the actuation frequency. (**e**) Experimental images of droplet capture and release with an SF-IDT, orange arrows indicate the activation of the SAWs. Figure (**d**,**e**) adapted with permission from Ref. [121]. Copyright 2017 by American Chemical Society.

For example, in the work proposed by Wang et al. [96], the authors developed an innovative microfluidic technique for on-demand droplet trapping and fusion, utilizing a DC electric field to selectively manipulate droplets within a chip. The device employs ITO electrodes strategically positioned near microwells to generate a non-uniform electric field, with the highest intensity located at the tip of the bottom electrode inside the microwell (see Figure 9a). When a droplet passes through this field, it becomes polarized, and the resulting dielectrophoretic force pushes it towards the region of highest field intensity, enabling its entrapment in the microwell. The dielectrophoretic force $F_{DEP}$ acting on the droplet is expressed as: $F_{DEP}=4\pi \epsilon r^{3}Re\left[f_{CM}\right]\nabla E^{2}$(where ϵ is the dielectric constant of the continuous medium, r is the radius of the droplet, $f_{CM}$ is the Clausius–Mossotti factor, and $\nabla E^{2}$ represents the gradient of the squared electric field intensity). The significant difference in dielectric permittivity between water and oil ${(\epsilon }_{H2O}\approx 81,\epsilon_{oil}\approx 2.5)$ ensures that the dielectrophoretic force is sufficiently strong to trap the droplets. The geometric design of the microwells and main channels ensures that the hydrodynamic resistance along the microwell path is greater than that of the main channel, preventing droplets from being passively trapped. Once a droplet is trapped, it can be fused with another selected droplet by applying a second electric field, as shown in the sequence depicted in Figure 9b(i–vi). Fusion enables the introduction of reagents or samples, allowing for controlled manipulation and analysis of chemical reactions. Thanks to these features, the proposed system offers high flexibility in droplet manipulation and enables in situ, real-time study of biochemical reactions without the need for high-speed cameras or other complex instrumentation. This approach is suited to numerous applications, such as enzyme kinetics, protein folding, and crystallization, making it a powerful tool for the development of high-precision lab-on-a-chip systems.

Conversely, another study proposed by Teste et al. [122], stands out from the previous ones by employing magnetic fields as the guiding principle. Specifically, the authors developed a microfluidic system that leverages magnetic rails for the selective manipulation of droplets (see Figure 9c). These magnetic rails are constructed by embedding ferromagnetic elements along microchannels, creating localized magnetic fields. When magnetic droplets—composed of liquid phases containing superparamagnetic nanoparticles—flow through the microchannel, they experience magnetic forces that guide their movement along the rails, enabling precise droplet handling and trajectory control.

One of the most compelling advantages of this technology lies in its ability to activate and deactivate the magnetic guidance on demand. By toggling the external magnetic field, individual droplets can be addressed in synchronization with their position, allowing the system to dynamically control each droplet’s path within the device. For instance, droplets can be diverted from their original trajectories and guided along the rails into specialized trapping structures. These traps consist of out-of-plane features designed to increase hydrodynamic resistance, diverting the main flow around the structures and preventing unintentional droplet capture in the absence of a magnetic field.

By activating the magnetic field as a droplet passes over a specific rail, the magnetic confinement force—which can range from 50 to 400 nN, depending on the applied current—overcomes the hydrodynamic drag forces and guides the droplet into the trap. Once immobilized, the magnetic field can be turned off, with the droplet retained in a stable position due to the trap geometry and flow-induced forces. This process enables the sequential trapping and parking of droplets in designated traps, while maintaining precise control over their positions. Additionally, the flow direction can be reversed to release droplets from the traps for collection or reintroduction into the main flow stream. The system demonstrates versatility in its ability to perform additional operations, such as selective droplet merging. After immobilizing a set of droplets in the traps, a second series of droplets can be introduced and guided to the same traps for coalescence. Droplet merging occurs when the flow velocity is increased beyond a threshold value, generating sufficient hydrodynamic force to overcome the stabilization provided by surfactants. The merged droplets can mix passively within seconds due to diffusion, or mixing can be actively enhanced using magnetic beads within the droplets by periodically toggling the magnetic field.

While the system offers high precision and flexibility, its throughput is currently limited by the droplet speed, as the magnetic rails must remain active throughout the droplet’s trajectory until it is stored in a trap. Nonetheless, the system achieves a throughput of approximately four droplets per minute under typical operating conditions, with the potential for faster processing rates through higher flow velocities and stronger magnetic forces.

This innovative approach not only provides robust control over droplet trapping and manipulation, but also integrates seamlessly with existing microfluidic architectures. Its ability to selectively sort, transport, release, and merge droplets with high accuracy makes it a promising tool for high-throughput biochemical analyses, single-cell studies and reaction monitoring in lab-on-a-chip systems.

Jung et al. introduced a novel acoustofluidic approach for droplet trapping and release, employing surface acoustic waves (SAWs) integrated into a microfluidic system with passive microwell traps (see Figure 9d) [121]. SAWs, known for their label-free, non-contact, and biocompatible nature [123,124,125], have long been utilized in microfluidic applications like droplet merging [126], sorting [127], steering [128], and splitting [129]. In this work, Jung et al. harnessed the acoustic radiation force (ARF) generated by SAWs to precisely manipulate droplets, capturing them in or releasing them from microwells with high accuracy.

The microfluidic device features a piezoelectric substrate (LiNbO_3_) paired with a PDMS microchannel. The SAWs are produced using a slanted finger interdigitated transducer (SF-IDT), capable of focusing acoustic energy into a narrow beam of ~100 µm. By tuning the frequency of the applied signal, the position of the SAW beam can be adjusted dynamically. This enables the controlled capture and release of droplets by aligning the acoustic force with the microwell location.

The microwells themselves are designed with bottleneck-shaped entrances, ensuring droplets remain securely trapped, resistant to external disturbances such as pressure fluctuations or continuous phase fluid flow. When a droplet is guided into a microwell, the ARF overcomes hydrodynamic drag, immobilizing the droplet with high precision (see Figure 9e). Releasing the droplet is as simple as shifting the SAW beam away from the microwell, allowing it to re-enter the fluid stream.

This system demonstrates exceptional control over droplet behavior, combining passive structural trapping with active acoustic manipulation. The integration of SAWs enhances the versatility of the platform, making it suitable for applications requiring precise droplet control, such as biochemical assays, single-cell analysis, and high-throughput microfluidic experiments.

After examining some of the most prominent works in the current literature, we can conclude that active trapping techniques represent a significant advancement in microfluidic technologies. By leveraging external forces such as electric, magnetic, and acoustic fields, these methods achieve unparalleled precision and adaptability in droplet manipulation. Their ability to integrate seamlessly with passive systems allows for a synergistic approach, combining natural fluid dynamics with externally induced control. These innovations underscore the transformative potential of active trapping methods, paving the way for groundbreaking applications in high-throughput biochemical analyses, single-cell studies, and complex reaction monitoring.

#### Comparative Assessment of Droplet Trapping

**Figure 10:**
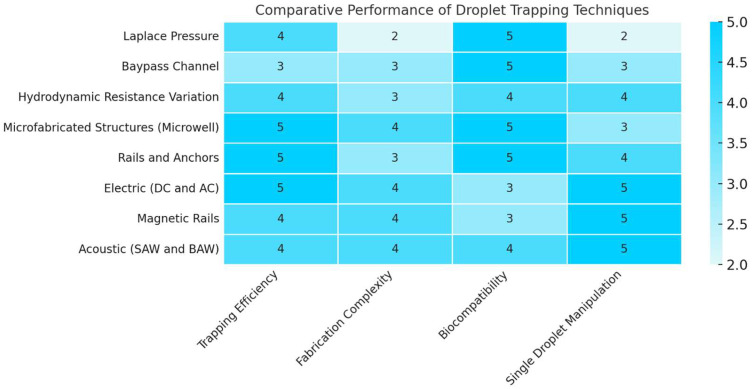
Comparative assessment of droplet trapping techniques based on four evaluation criteria: trapping efficiency, fabrication complexity, biocompatibility, and single-droplet manipulation capability. Passive methods—such as Laplace pressure traps, bypass channel architectures, hydrodynamic resistance modulation, and microwell-based structures—are compared alongside active techniques including electric (DC and AC), magnetic rails, and acoustic (SAW and BAW) systems. Scores range from 1 (poor) to 5 (excellent) and reflect aggregated performance across representative studies.

Figure 10 presents a comparative analysis of major droplet trapping methods—both passive and active—evaluated across four key criteria: trapping efficiency, fabrication complexity, biocompatibility, and single-droplet manipulation.

Passive approaches such as Laplace pressure, bypass channels, and hydrodynamic resistance variations offer high biocompatibility and simplicity, making them ideal for long-term culture or drug testing. However, they often lack fine control and reversibility. Microwell structures and rails and anchors improve positional stability and compatibility but still depend heavily on fixed geometries.

Active techniques—electric (DC/AC), magnetic rails, and acoustic (SAW/BAW)—enable precise, on-demand droplet control and are better suited for dynamic manipulation. Despite higher complexity, they offer advantages in scalability, programmability, and integration into automated workflows.

From a translational perspective, passive methods remain valuable for low-cost, robust systems in biological and diagnostic applications. Active methods, meanwhile, are increasingly adopted in industrial settings—particularly in high-throughput screening, single-cell assays, and lab-on-chip diagnostics—where precision, automation, and scalability are essential.

## 3. On-Demand Droplet Sorting Techniques

The ability to systematically sort and categorize droplets is a cornerstone of droplet-based microfluidic systems, playing a critical role in the realization of various lab-on-a-chip applications. The efficient sorting and integration of droplets into fluidic workflows are essential to harness their full potential for biological studies, chemical assays and diagnostic applications. Recent advancements have pushed the boundaries of droplet sorting, achieving sorting rates up to the kilohertz range, yet challenges remain in terms of precision, scalability, and adaptability.

**Figure 11:**
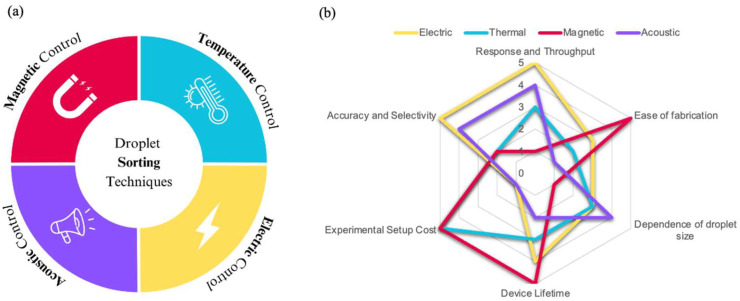
Examples of droplet sorting using active techniques. (**a**) Overview of the main sorting strategies and their categorization. (**b**) The diagram compares various methods for sorting droplets based on six performance criteria: (i) response time and throughput, (ii) ease of fabrication, (iii) cost of setup, (iv) accuracy and selectivity, (v) device lifetime, and (vi) dependency on droplet size. Each criterion is rated on a scale from one to five, with five indicating the highest performance.

In this section, we narrow our focus to active sorting techniques, deliberately excluding passive methods. While passive sorting relies purely on the inherent fluid dynamics and device geometry [95,130,131], active methods leverage external physical forces, such as electric, magnetic, acoustic, and thermal fields, to achieve on-demand control. This focus is motivated by the superior precision, flexibility, and adaptability offered by active approaches, which are indispensable for complex and high-throughput applications. Figure 11a offers a comprehensive overview of the various sorting strategies discussed in the following sections. These strategies are categorized based on their actuation mechanisms: electric, acoustic, magnetic, and thermal. Additionally, six key performance metrics associated with each control type are considered in Figure 11b: (i) response time and throughput, (ii) fabrication simplicity, (iii) experimental setup cost, (iv) accuracy and selectivity, (v) device longevity, and (vi) sensitivity to droplet size. For a more in-depth analysis, readers are referred to the excellent review by Xi et al. (2017) on the main active sorting techniques [132].

Active sorting techniques excel in their ability to dynamically respond to feedback, enabling the selective discrimination of droplets based on size, content, or physical properties such as optical, electric, or magnetic signatures. These attributes make active methods particularly suited to applications requiring high specificity, such as single-cell studies, rare event detection, or multi-step biochemical workflows. Furthermore, by concentrating on active mechanisms, we aim to highlight the cutting-edge innovations and future possibilities in droplet sorting, emphasizing their transformative impact on microfluidic technologies.

### 3.1. Electric Control

Electric sorting methods have seen significant advancements over the past decade, emerging as a highly effective and precise alternative to passive sorting approaches. Unlike passive methods that rely on hydrodynamic properties like size or shape, electric sorting enables on-demand control by exploiting the electrical properties of droplets. This approach achieves high throughput and accuracy through rapid electric detection and actuation, often operating on time scales as short as microseconds.

The effectiveness of electric sorting depends on the presence of free charges within the droplet or distinct differences in conductivity and permittivity between the dispersed droplet and the carrier fluid. These differences generate interfacial or bulk stresses that drive droplet dynamics under electric forces. While this requirement may limit the choice of compatible liquids, the inherent immiscibility of most microfluidic systems often ensures sufficient electrochemical contrast.

In 2010, Guo et al. developed an innovative droplet sorting technique based on the phenomenon of droplet self-charging [133]. In this approach, water-in-oil droplets are generated using a flow-focusing configuration, with a lateral oil injection used to adjust the distance between adjacent droplets. As the droplets pass through an electric field produced by two oppositely charged indium tin oxide (ITO) electrodes placed alongside the main flow channel, they acquire a positive charge. This field induces a deflection of the droplets, which are directed to different collection branches based on their charge.

Although the exact mechanism of the self-charging phenomenon remains unclear, it is hypothesized to result from ion transformation or induced charging between the aqueous droplet and the oil phase. This phenomenon leads to the droplets becoming positively charged and subsequently deflected towards the negative side. By switching the polarization of the electrodes, droplets can be sorted into different branches, making the technique highly adaptable.

The device consists of a droplet generator, an electric controller and a collector. The application of an electric field through DC pulses allows for the deflection of the charged droplets towards the desired collector, achieving sorting speeds of up to 100 Hz. This method has proven successful for sorting single cells encapsulated in alginate droplets, providing rapid separation (15 ms per droplet) and high precision.

One of the key advantages of this technique is its lack of pre-charging of the droplets, simplifying the process and improving efficiency. The proposed hydrodynamic model describes the movement of the droplets under the influence of the electric field, enabling precise and accurate sorting. This approach has significant potential for applications in biotechnology, such as single-cell analysis, cell lysis, and other microfluidic operations, due to its simplicity, speed, and adaptability.

**Figure 12:**
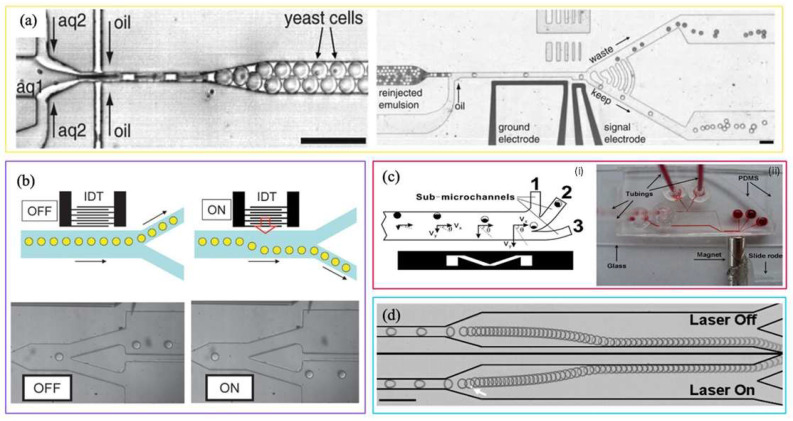
Examples of droplet sorting using active techniques. (**a**) Modules of the ultrahigh-throughput microfluidic screening platform. Figure (**a**) reprinted with permission from Ref. [134]. Copyright 2010 by National Academy of Sciences. (**b**) Schematic of the hybrid PDMS–SAW chip: the droplet generation unit and a branched PDMS channel coupled to a SAW device. With SAW power off, all drops flow through the upper channel due to lower flow resistance. When SAW power is on, acoustic streaming from the IDT (red arrow) redirects droplets to the lower channel [127]. (**c**) (**i**) Schematic diagram of the droplet velocity component. V_x_ and V_y_ are the droplet velocity components in the x-direction and y-direction, respectively. (**ii**) Schematic representation of a superparamagnetic droplet deflected in a microchannel. Magnetic and drag forces act in opposite directions along the y-axis. Closer to the magnet, the stronger magnetic force pulls nanoparticles downward, creating a white (water) top and orange (nanoparticle) bottom in the droplet. [145]. (**d**) Superposition of successive frames illustrating drop switching by local thermocapillary actuation; the arrow indicates the laser location observed by the fluorescence of the water–dye solution [151].

Conversely, Agresti et al. introduced a groundbreaking sorting method using dielectrophoresis (DEP) and alternating current (AC) electric fields, demonstrating its potential for high-throughput screening in microfluidics (see Figure 12a) [134]. This technique is based on a bifurcation microfluidic device designed to manipulate droplets containing biological entities, such as yeast cells, with great precision and efficiency.

The device employs a bifurcation junction, where two branches are set to have different hydrodynamic resistances. In the absence of an electric field, droplets naturally flow into the branch with lower resistance. When sorting is required, AC electric fields are applied through electrodes placed near the junction. The dielectrophoretic forces generated by the electric field cause the droplets to be directed into the desired collection channel. This AC-based approach offers a clear advantage over traditional direct current (DC) methods by reducing the risk of droplet coalescence, a common issue in DC-based sorting systems. By adjusting the frequency and amplitude of the AC signal, the system can be finely tuned for precise droplet control, ensuring they are sorted without merging, thus maintaining their integrity.

One of the most significant aspects of this method is its ability to perform sorting at high speeds. The system can process droplets at rates of thousands per second, enabling ultrahigh-throughput screening. In their study, Agresti et al. demonstrated its capability by screening a library of enzyme variants—10^8^ reactions—in only 10 h using less than 150 μL of total reagent volume. This efficiency represents a 1000-fold increase in speed and a 10-million-fold reduction in reagent consumption compared to conventional robotic systems. Such speed and efficiency make this approach ideal for large-scale screenings, where time and cost constraints are critical.

The implications of this technology are profound, especially in the realm of biotechnology. Its ability to sort droplets at high speeds with such precision opens new doors for directed enzyme evolution, biochemical screenings, and other high-throughput applications. In the study, the authors used the platform to identify horseradish peroxidase mutants with activities more than 10 times faster than the parent enzyme. By allowing researchers to explore vast libraries of biological or biochemical reactions in a fraction of the time required by traditional methods, this technology has the potential to revolutionize fields like synthetic biology, molecular engineering, and drug discovery, offering a much-needed breakthrough in modern biotechnology.

### 3.2. Acoustic Control

Acoustic control is a promising method for droplet sorting, particularly due to its high biocompatibility. It allows for the manipulation of biological samples, such as cells, encapsulated in droplets, maintaining viability even after long exposure to acoustic fields. Acoustic waves are generated by piezoelectric actuators [135] and can be classified into two types: surface acoustic waves (SAW) [123,136], which propagate along the surface, and bulk acoustic waves (BAW) [136], which travel through the medium itself.

These waves give rise to acoustic streaming (fluid flow induced by sound waves) [137] and acoustic radiation force (force on suspended objects) [138], both of which are utilized for sorting droplets. The effectiveness of acoustic sorting depends on the size of the droplets and the physical properties of the surrounding liquid. Optimizing the frequency and geometrical design of the microfluidic system is crucial to achieve efficient sorting.

#### 3.2.1. SAWs

Surface acoustic waves (SAWs) are a type of wave that propagates along the surface of a piezoelectric material, such as lithium niobate (LiNbO_3_). These waves are generated by an interdigitated transducer (IDT), which consists of two comb-like electrodes placed on the surface of the substrate [139]. When an alternating current (AC) is applied to the IDT, it generates an oscillating displacement of the substrate, creating the SAWs.

The frequency of the SAWs is determined by the dimensions of the electrodes and can range from 10 MHz to 1000 MHz, allowing for the manipulation of droplets and particles of different sizes. SAWs can be classified into two main types based on their behavior: traveling surface acoustic waves (TSAWs) and standing surface acoustic waves (SSAWs). In TSAWs, the wave propagates in one direction, whereas in SSAWs, the wave oscillates in place, creating nodes and antinodes on the surface.

Franke et al. firstly demonstrated the use of traveling surface acoustic waves (TSAWs) for droplet sorting in microfluidic systems [127]. Their experimental setup featured a PDMS microchannel with a Y-junction placed on top of a LiNbO_3_ piezoelectric substrate. The Y-junction was designed with the upper branch having a lower flow resistance, allowing droplets to flow into this branch when the interdigitated transducer (IDT) was off. When the IDT was activated at its resonance frequency of around 140 MHz, it generated a Rayleigh wave that propagated along the substrate, coupling acoustic energy into the fluid. The propagating TSAW partially radiated into the microchannel, causing the droplets to be deflected in the direction of wave propagation, ultimately directing them toward the lower branch of the Y-junction (See Figure 12b). This effect was primarily attributed to acoustic streaming, where the high-frequency wave induces bulk fluid motion, pushing the droplets. However, since the droplets were 20 μm in size (with κ ≈ 5.9), it was suggested that the acoustic radiation force—resulting from the interaction between the TSAW and the droplets’ acoustic impedance mismatch—could also play a role in their deflection.

#### 3.2.2. BAWs

Unlike surface acoustic waves (SAWs), which propagate along a material’s surface, bulk acoustic waves (BAWs) are ultrasonic waves that travel through the material’s interior [83,140]. These waves are typically generated by bulk piezoelectric transducers, differing from the interdigitated transducers used in SAW devices. To implement BAWs in microfluidic systems, an acoustic resonator is created by placing a piezoelectric transducer underneath the fluidic channel. When the transducer is excited at its resonant frequencies, it generates a standing wave field across the channel. The resonance frequencies of the system can be expressed as $f_{res}^{n}=c_{f}n/2w$, where w is the channel width and n=1,2,3… denotes the order of harmonics. This formula indicates that the system can generate a standing acoustic field across the channel width. In this setup, materials with high acoustic impedance, such as silicon or glass, are often used for constructing the device. The large difference in acoustic impedance between the fluid and the substrate results in the reflection of most acoustic energy back into the fluid, creating a strong standing wave that can manipulate particles within the fluid. This technique has been effectively used in various applications, including the separation of particles and biological cells, within microfluidic devices. The standing wave field induced by the BAWs applies forces on suspended objects, sorting them based on their size and acoustic properties.

Lee et al. developed an innovative ultrasonic method for sorting lipid droplets in a microfluidic channel using a 30 MHz ultrasound beam [141]. The setup utilized a PDMS microfluidic channel with a bifurcation, where the main advantage of this configuration stems from the similar acoustic impedance between PDMS and water. This similarity minimizes sound reflection at the PDMS–water interface, ensuring a more efficient transmission of acoustic waves into the fluid. To enhance the transmission of ultrasonic waves into the microfluidic channel, both the acoustic transducer and the microfluidic device were immersed in a water bath.

The system operates in two phases: acoustic sensing and sorting. Initially, short sensing pulses from a lithium niobate (LiNbO_3_) transducer are transmitted to probe individual droplets. The integrated backscatter (IB) coefficient, which is determined by analyzing the echo signals from each droplet, is used to distinguish between droplets of different sizes. When the IB coefficient of a 100 μm droplet is detected, the system switches to sorting mode. A burst of 2000 cycles of 30 MHz sinusoidal signals is then applied, creating an acoustic radiation force that pushes the droplet towards the desired channel branch.

This approach demonstrated high sorting efficiencies: 99.3% for 50 μm droplets and 85.3% for 10 μm droplets. The authors noted that the radiation force likely originates from the traveling wave, given the limited reflection at the PDMS–water interface. Moreover, the size of the lipid droplets (100 μm) corresponds to a κ ≈ 6.3, indicating that the acoustic radiation force generated by the traveling wave effectively manipulates droplets of this size.

### 3.3. Magnetic Control

Magnetic droplet manipulation offers a wireless control method that is useful for chemical and biological applications, especially when handling droplets containing biological reagents or chemical samples. The technique is relatively simple to implement, but requires magnetic materials in the fluid, which can affect chemical and biological compatibility. Typically, ferrofluids [142] or magnetic beads [143] are used for sorting, as they interact with the applied magnetic field. While effective, magnetic manipulation has a slower response compared to other methods, with switching frequencies usually in the low Hertz range. The magnetic actuation can be achieved using either electromagnets, which allow adjustable field strength, or permanent magnets, which are often preferred due to their lower risk of generating harmful heat.

Surenjav et al. introduced a droplet sorting method leveraging an electromagnet in conjunction with a microfluidic platform [144]. This system utilized ferrofluid as the continuous phase, with water as the dispersed phase to form a stable gel emulsion without the need for additional surfactants. Ferrofluid, a colloidal liquid containing superparamagnetic nanoparticles, responds to magnetic fields and was instrumental in enabling magnetic sorting. The gel emulsion was generated using either step-emulsification or T-junction cross-shear methods, leading to monodisperse droplets.

Sorting was achieved by applying an inhomogeneous magnetic field via an electromagnet placed near the microchannel. This field caused the ferrofluid to flow toward regions of higher magnetic strength, forming a ferrofluid plug in the lower branch of a bifurcation. This plug acted as a stop valve, redirecting droplets to the upper channel. This approach demonstrated precise control over droplet routing and highlighted the versatility of ferrofluid-based manipulation for active droplet processing in microfluidic systems.

For example, in the context of permanent magnets, Zhang et al. demonstrated an efficient magnetic droplet sorting method utilizing superparamagnetic magnetite (Fe_3_O_4_) nanoparticles dispersed in water droplets within an oil carrier phase [145]. The system consisted of a PDMS microfluidic channel with three outlets, each with different flow resistances (see Figure 12c(i,ii)). In the absence of a magnetic field, droplets naturally flowed into the channel with the least resistance. When a permanent magnet was placed near the channel, the nanoparticles in the droplets became magnetized, inducing a magnetic force that counteracted the Stokes drag and deflected droplets into designated sub-channels.

The deflection depended on the position of the magnet relative to the bifurcation, with configurations enabling full, partial, or zero deflection into specific outlets. The sorting efficiency remained high across various flow rates, droplet sizes, and nanoparticle concentrations, achieving sorting rates of approximately 10 droplets per second. This method’s simplicity and compatibility with biomolecules make it suitable for applications in drug delivery, immunoassays, and cell sorting.

### 3.4. Thermal Control

The temperature-based droplet sorting method leverages the thermocapillary effect to manipulate droplet movement on the microscale [146,147]. This effect arises when temperature gradients induce variations in surface tension, generating forces that direct droplets along desired paths [148]. Two common techniques to create these temperature gradients are resistive heating and focused laser beams.

Significantly, although niche, thermal control methods are useful when other droplet sorting approaches fail to meet specific system requirements, particularly in cases where precise temperature control is critical.

#### 3.4.1. Resistive Heating

Microheaters, often integrated with temperature sensors, provide precise, localized heating at specific points within the microfluidic system. This approach allows for real-time temperature monitoring, a crucial feature for applications like the polymerase chain reaction (PCR) or other temperature-sensitive biological processes. However, it requires a careful alignment of the heating elements with the microchannel [149].

#### 3.4.2. Laser-Based Heating

Focused laser beams offer greater flexibility, enabling the manipulation of droplets across different locations without physical contact [150]. While this method provides spatial adaptability, it typically necessitates external temperature calibration to correlate beam intensity with the resulting thermal gradient.

An example of laser-based heating for droplet sorting is provided by Robert de Saint Vincent et al., who utilized thermocapillary stresses to deflect droplets in a microfluidic setup [151]. In their study, droplets were introduced into a chamber with two asymmetric outlets, where a focused laser beam generated localized temperature gradients at the droplet interface. These gradients created thermocapillary forces—driven by the Marangoni effect—that deflected droplets toward the upper outlet (see Figure 12d). The researchers reported 100% switching efficiency for droplets moving at velocities up to 1.3 cm/s, highlighting the method’s potential for high-throughput droplet sorting. This approach is particularly advantageous in lab-on-a-chip applications due to its non-invasive nature, high precision, and versatility in handling diverse droplet types.

## 4. Conclusions and Perspective

In recent years, the field of droplet manipulation and trapping in microfluidic systems has undergone a significant transformation, thereby expanding the boundaries of research and opening new perspectives for applications in biology, chemistry, and precision medicine. In this review, a detailed analysis of droplet generation, trapping, and sorting strategies is presented, distinguishing between passive and active approaches. Passive methods are distinguished by their simplicity of fabrication and the absence of external actuators, while active methods offer dynamic and precise control over droplets through the use of electric, magnetic, acoustic, and thermal forces.

Despite significant advancements in the field, several challenges remain to be addressed. One such challenge is the integration of multiple manipulation techniques within a single microfluidic device, a crucial step toward improving the efficiency and versatility of lab-on-a-chip systems. Additionally, optimizing the biocompatibility of materials used in these devices is essential to prevent unwanted effects on cells and biomolecules, thereby ensuring the reliability of experimental results.

Another unresolved challenge pertains to the reproducibility of droplet generation, trapping, and sorting processes, which is crucial for precise and repeatable control, ensuring reliable and comparable results, especially in high-precision applications such as clinical diagnostics or drug screening. Currently, variability in operating parameters (e.g., pressure, surface tension, or temperature) can significantly influence droplet size, shape, and distribution, compromising reproducibility.

To address this issue, real-time monitoring techniques and automated feedback systems are being developed to dynamically regulate process parameters, reducing fluctuations and improving result consistency [152]. Looking ahead, there is a growing interest in automation and artificial intelligence applied to microfluidics. The employment of machine learning algorithms has the potential to enhance the precision and reproducibility of droplet manipulation in real time [153,154]. Simultaneously, advancements in 3D printing technologies for the rapid fabrication of customized microfluidic devices promise to lower costs and accelerate research processes [155]. Another key area of interest is the integration of on-chip biosensors, which will enable real-time monitoring of reactions within droplets, with groundbreaking implications for clinical analysis and the development of personalized medicine.

Finally, emerging applications such as digital microfluidics and the use of smart materials for droplet control could redefine the landscape of the field, opening new frontiers for fluid manipulation at the microscale [156,157]. In this rapidly evolving context, interdisciplinarity between engineering, physics, biology, and materials science will be essential to tackling future challenges and fully exploiting the potential of microfluidic technologies. The synergy between these fields is poised to transform microfluidics into an increasingly powerful and versatile tool, with significant impacts on both scientific research and industrial applications.

## Figures and Tables

**Table 1:** Dimensionless numbers describing hydrodynamic regime of droplet generation inside microchannel; μ is the fluid viscosity, u is the average fluid velocity, γ is the interfacial tension between two phases, d_h_ is the characteristic length of micro-channel, ρ is the fluid density, and g is the gravitational acceleration.

Dimensionless Number	Formula	Physical Significance
Capillary number (Ca)	$Ca=\frac{\mu u}{\gamma }$	$\frac{Viscous\;force}{Interfacial\;tension}$
Reynolds number (Re)	$Re=\frac{\rho ud_{h}}{\mu }$	$\frac{Inertial\;force}{Viscous\;force}$
Weber number (We)	$We=\frac{\rho u^{2}d_{h}}{\gamma }$	$\frac{Inertial\;force}{Interfacial\;tension}$
Bond number (Bo)	$Bo=\frac{\rho gd_{h}}{\gamma }$	$\frac{Gravitational\;force}{Interfacial\;tension}$

**Table 2:** A summary of key works related to the context of rail and anchor mechanisms.

Main Technology	Innovations/Contributions	Applications	Key Point (Rails and Anchors)	Ref (s)
Microfluidic system for co-encapsulation of two aqueous phases in droplets.	This work combines both passive and active fluid manipulation. Floating traps and a rail system are employed for droplet filtering and pairing. Additionally, electrocoalescence is used for merging with 100% trapping efficiency.	This system enables the generation of dual-content droplets for studying cell–cell interactions and particle–cell dynamics, and creating libraries of droplets with different aqueous compositions.	This system incorporates a floating trap array (FTA) for the efficient pairing of droplets, with optimized rail dimensions based on droplet height and size (e.g., 35 µm).	[114]
Microfluidic platform for 3D cell cultures and spheroid formation.	This platform enables the geometric manipulation of droplets for guidance and stabilization. Spheroids are suspended in agarose and solidified at low temperatures and can be selectively recovered using a focused IR laser.	Used for high-density 3D cell culture, enabling long-term monitoring and analysis of spheroids for stem cell, organ-on-chip, and oncology research.	Geometric anchors help stabilize and spontaneously position the droplets. Additionally, geometric manipulation rails create confinement gradients for droplet movement.	[115]
Droplet microfluidics for functional screening and real-time monitoring of TCR T cell activation.	This system enables single-clone tracking with 100% sorting specificity. It uses an inverted floating droplet array (iFDA) for trapping and observing droplets, while sorting is achieved through UV laser-induced cavitation.	Used for functional screening of TCR T cell clones, real-time tracking of T cell activation, and sorting clones for downstream analysis such as RT-PCR and sequencing.	The use of an inverted floating droplet array (iFDA) with 1296 traps allows the precise placement of droplets. The parameters for the traps, including height and diameter, are optimized for effective sorting.	[116]
Multi-droplet clustering device with sequential trapping and storage.	This technology allows for droplet shape manipulation, enabling trapping in both forward and reverse flows. A 10 × 12 array platform is used for clustering and storage, while buoyancy and interfacial energy effects help store droplets in dedicated chambers.	Suitable for clustering droplets containing different samples for complex chemical and biological reactions, and for studying clustered droplet-based reactions.	This system relies on sequential trapping for droplet clustering, with guiding rails ensuring accurate positioning in storage chambers. It combines interfacial energy and buoyancy effects for permanent droplet storage.	[117]
Dual-layer microfluidics for floating droplet manipulation.	A dual-layer device is used to manipulate and analyze droplets at ultrahigh throughput. Buoyancy helps trap droplets in hundreds of thousands of wells. The system also allows for the digital quantification of analytes via droplet counting, and real-time analysis of fluorescein diffusion.	This platform is applied for high-throughput droplet analysis, the quantification of analytes, and real-time observation of diffusion processes, such as enzymatic reactions.	The floating droplet array (FDA) traps droplets in high-density wells. Droplet size is controlled for precise clustering within individual wells, typically one to four droplets per well.	[118]

**Table 3:** A summary of the key works in the context of droplet trapping using active techniques.

Active Technique	Description	Applications	Key Features	Ref(s)
Electrostatic potential wells—hybrid (microfluidic + electrostatics)	This paper proposes a new technique for manipulating droplets in microchannels using co-planar electrodes to generate electrostatic potential wells. Electrostatic forces enable operations such as trapping and releasing droplets, based on the balance between electrostatic and hydrodynamic forces.	Droplet manipulation (trapping, release, and guiding) in microchannels and lab-on-a-chip applications	This technique uses co-planar electrodes separated by a narrow gap to generate an electrostatic field for confining and manipulating droplets. The intensity of the electrostatic force is adjustable via the applied voltage, allowing for the modification of the trapping force. Both trapping forces and hydrodynamic forces can be independently adjusted, allowing the precise manipulation of droplets of varying sizes. Applications include the separation, guiding, and manipulation of droplets with cells, which can then be analyzed through techniques such as surface plasmon resonance (SPR).	[119]
On-demand trapping and fusion of microfluidic droplets—microfluidics (DC electric field)	This paper presents a microfluidic structure using a DC electric field to trap selected droplets in a micro-reservoir and fuse a droplet with the trapped one. This process can introduce reagents into droplets and enable real-time monitoring of chemical reactions without the need for high-speed cameras.	Droplet manipulation for on-chip experiments, chemical and biological studies, rapid reactions, enzyme kinetics, protein folding, and crystallization	This technique uses a dielectrophoretic (DEP) electric field to trap and fuse microfluidic droplets. Devices are made from PDMS and use silicon oil and water as fluid phases. The application of a DC electric field generates a non-uniform electric field that allows precise droplet manipulation. The design enables in situ monitoring of chemical reactions within droplets, facilitating real-time studies without high-speed cameras.	[96]
Surface acoustic waves (SAW)—acoustofluidics	This work presents an acoustofluidic platform that uses surface acoustic waves (SAW) for easy droplet trapping and release within micro-reservoirs in a microfluidic channel. Acoustic waves push or pull droplets toward the micro-reservoir, enabling selective trapping or release.	Droplet manipulation in microchannels, bioanalysis applications, droplet separation, and fusion	This technique uses the acoustic radiation force (ARF) generated by SAWs to trap and release droplets in micro-reservoirs. The platform is made using a piezoelectric substrate (LiNbO_3_) and a PDMS microfluidic channel. The device is designed to manipulate droplets precisely by controlling the frequencies of the acoustic waves, which move the SAW beam position and modulate droplet release or trapping. The system is highly biocompatible and non-invasive.	[120]
Ferromagnetic rails and magnetic force—magnetic (magnetofluidics)	This work proposes a new technology combining ferromagnetic rails and magnetic droplets for precise droplet manipulation in microfluidic devices. When the magnetic field is activated, magnetic droplets follow a determined path, while hydrodynamic drag forces act as transport forces.	Magnetic droplet separation and manipulation, chemical and biological studies, and parallel enzymatic reactions	This technique uses ferromagnetic rails and a magnetic field to manipulate magnetic droplets in a microfluidic device. Manipulation occurs through a combination of hydrodynamic and magnetic forces that guide droplets along a predefined path. The magnetic field can be turned on and off to select and direct individual droplets toward parking areas or perform droplet fusion operations. The technology provides high temporal and spatial resolution for operations such as fusion, parking, and droplet separation.	[121]
Bulk acoustic waves (BAW)—acoustofluidics	This acoustofluidic platform uses bulk acoustic waves for non-contact trapping of cell-laden hydrogel droplets. Acoustic waves generate a stationary field that traps droplets without physical contact. The system is compatible with optical microscopy, enabling operations like perfusion and reagent addition.	Droplet manipulation with cells, biological and biochemical studies, real-time analysis, and long-term monitoring	This system uses bulk acoustic waves generated by a piezoelectric transducer to trap hydrogel droplets with cells in a continuous fluid configuration. Droplets are positioned in a well-defined location within the capillary, enabling perfusion and analysis through optical microscopy. The technique is label-free, gentle, and well-suited for biological applications.	[122]

## Abbreviations

The following abbreviations are used in this manuscript:

DBM	Droplet-based microfluidics
Ca	Capillary number
We	Weber number
Re	Reynolds number
Bo	Bond number
DEP	Dialectrophoresis
EWOD	Electrowetting-on-dielectric
DLD	Deterministic lateral displacement
PDMS	Polydimethylsiloxane
SAW	Surface acoustic waves
BAW	Bulk acoustic waves
PCR	Polymerase chain reaction
iFDA	Inverted floating droplet array
